# Plasma Membrane Phosphatidylinositol-4-Phosphate Is Not Necessary for Candida albicans Viability yet Is Key for Cell Wall Integrity and Systemic Infection

**DOI:** 10.1128/mbio.03873-21

**Published:** 2022-02-15

**Authors:** Rocio Garcia-Rodas, Hayet Labbaoui, François Orange, Norma Solis, Oscar Zaragoza, Scott G. Filler, Martine Bassilana, Robert A. Arkowitz

**Affiliations:** a Université Côte d’Azur, CNRS, INSERM, Institute of Biology Valrose, Nice, France; b Université Côte d’Azur, CCMA, Nice, France; c Institute for Infection and Immunity, Lundquist Institute for Biomedical Innovation at Harbor-UCLA Medical Center, Torrance, California, USA; d Mycology Reference Laboratory, National Centre for Microbiology, Health Institute Carlos III, Majadahonda, Madrid, Spain; e David Geffen School of Medicine at UCLA, Los Angeles, California, USA; Duke University

**Keywords:** *Candida albicans*, phosphatidylinositol phosphates, cell wall, filamentous growth, opportunistic fungi, phospholipids, virulence

## Abstract

Phosphatidylinositol phosphates are key phospholipids with a range of regulatory roles, including membrane trafficking and cell polarity. Phosphatidylinositol-4-phosphate [PI(4)P] at the Golgi apparatus is required for the budding-to-filamentous-growth transition in the human-pathogenic fungus Candida albicans; however, the role of plasma membrane PI(4)P is unclear. We have investigated the importance of this phospholipid in C. albicans growth, stress response, and virulence by generating mutant strains with decreased levels of plasma membrane PI(4)P, via deletion of components of the PI-4-kinase complex, i.e., Efr3, Ypp1, and Stt4. The amounts of plasma membrane PI(4)P in the *efr3*Δ/Δ and *ypp1*Δ/Δ mutants were ∼60% and ∼40%, respectively, of that in the wild-type strain, whereas it was nearly undetectable in the *stt4*Δ/Δ mutant. All three mutants had reduced plasma membrane phosphatidylserine (PS). Although these mutants had normal yeast-phase growth, they were defective in filamentous growth, exhibited defects in cell wall integrity, and had an increased exposure of cell wall β(1,3)-glucan, yet they induced a range of hyphal-specific genes. In a mouse model of hematogenously disseminated candidiasis, fungal plasma membrane PI(4)P levels directly correlated with virulence; the *efr3*Δ/Δ mutant had wild-type virulence, the *ypp1*Δ/Δ mutant had attenuated virulence, and the *stt4*Δ/Δ mutant caused no lethality. In the mouse model of oropharyngeal candidiasis, only the *ypp1*Δ/Δ mutant had reduced virulence, indicating that plasma membrane PI(4)P is less important for proliferation in the oropharynx. Collectively, these results demonstrate that plasma membrane PI(4)P levels play a central role in filamentation, cell wall integrity, and virulence in C. albicans.

## INTRODUCTION

Phosphatidylinositol phosphates are minor components of cellular membranes that play an essential role during polarized growth. In particular, phosphatidylinositol-4-phosphate [PI(4)P] is found predominantly at the Golgi apparatus and plasma membranes, generated from the precursor PI which is synthesized at the cytosolic face of the endoplasmic reticulum (ER) (1). Type III PI4-kinases are found in virtually all eukaryotes, with *PIK1* in fungi being homologous to mammalian type IIIβ PI4-kinases and Stt4 to mammalian type IIIα PI4-kinases (2, 3). While all fungi appear to have Stt4 orthologs, *PIK1* orthologs are absent in some of them, including Aspergillus nidulans and Cryptococcus neoformans (4, 5), and it has been suggested that Stt4 can carry out *PIK1* essential functions in these fungi. Plasma membrane PI(4)P generated by Stt4 has been implicated in the control of membrane trafficking, lipid exchange, cell signaling, cytoskeleton organization, and cytokinesis. Stt4-type IIIα PI4-kinases are essential for viability in most fungi, including Saccharomyces cerevisiae and Schizosaccharomyces pombe (6–9); however, *stt4* mutants can be rescued by osmoremediation (6, 9).

Candida albicans is a major human fungal opportunistic pathogen that grows in both yeast and filamentous forms. The morphological transition between yeast and filamentous growth is important for its virulence and can be triggered by a range of stimuli (10–12). Using mutants in which the levels of phosphatidylinositol phosphates can be reduced revealed that Golgi PI(4)P and plasma membrane PI(4,5)P_2_ are critical for this transition (13, 14). Mutants with reduced levels of the plasma membrane PI-4-kinase Stt4 can nonetheless form germ tubes and grow invasively (14), raising the question of whether this pool of PI(4)P is critical for the yeast-to-hypha transition. In C. albicans (13), as in S. cerevisiae (15, 16), the Golgi apparatus and plasma membrane pools of PI(4)P are functionally distinct; i.e., Golgi PI(4)P does not substantially contribute to plasma membrane PI(4)P. A previous study suggested that the plasma membrane type IIIα PI4-kinase Stt4 was not essential for viability in C. albicans (17), raising the possibility that plasma membrane PI(4)P may be dispensable.

In yeast and mammalian cells, Stt4 is part of a complex comprising the membrane protein Efr3 and the scaffold protein Ypp1 (TTC7 in mammals). Efr3 and Ypp1 are required for both targeting of Stt4 to the plasma membrane and PI-4-kinase activity (18–20). Specifically, Efr3 is critical for the plasma membrane association of the PI-4-kinase complex, and Ypp1/TTC7 has been shown to bind directly both Efr3 and Stt4 (18, 19), suggesting a scaffolding function. In S. cerevisiae, both Ypp1 and Efr3 are essential for viability (18, 21, 22), whereas in S. pombe, only Ypp1 is essential for viability (8). In conditional *ypp1* and *efr3* S. cerevisiae mutants, cellular PI(4)P levels were reduced approximately 2-fold (18, 20, 22).

To investigate the importance of plasma membrane PI(4)P in C. albicans hyphal growth and virulence, we generated *efr3* and *ypp1* deletion mutants, in addition to *stt4*. These mutants were all viable and had different levels of plasma membrane PI(4)P. Surprisingly, C. albicans cells with little to no plasma membrane PI(4)P remained viable and could proliferate by budding growth. Furthermore, our results indicate that plasma membrane PI(4)P is critical for hematogenously disseminated candidiasis (HDC), with a *ypp1* mutant exhibiting reduced virulence and an *stt4* mutant causing no lethality, consistent with their plasma membrane PI(4)P levels. Our analyses of the cell wall suggest that the dramatic reduction in virulence in HDC is, in part, due to an unmasking of cell surface β(1,3)-glucan.

## RESULTS

### The nonessential Stt4 PI-4-kinase complex is critical for invasive filamentous growth and cell wall integrity.

Previously, we generated strains in which the expression of *STT4* could be repressed with the addition of doxycycline (14). In the presence of this repressor, there was approximately a 10-fold decrease in *STT4* transcript levels, and budding growth was similar to that of the wild type. When *STT4* was repressed, this mutant was able to undergo invasive filamentous growth in response to serum, yet invasive filaments emanating from mutant colonies were ∼4-fold shorter than those of wild-type and complemented strains. Upon repression of *STT4* in liquid medium containing serum, the cells elongated with protrusions that were roughly one-third the length of wild-type cells after 2 h at 37°C. However, under these repression conditions, the *stt4* mutant still expressed *STT4* and plasma membrane PI(4)P was still detected (14). We also generated a DAmP (decreased abundance by mRNA perturbation) allele (23) in C. albicans (24), by constructing strains in which one copy of *STT4* was deleted and a dominant nourseothricin resistance marker (*SAT1*) was integrated just 3′ of the *STT4* stop codon. These DAmP mutants had between 2- and 4-fold reduction in *STT4* transcript levels compared to a wild-type strain, yet filamentous growth was indistinguishable from that of the wild type in liquid and on solid media containing fetal calf serum (Fig. S1). More recently, an *stt4* deletion mutant was isolated in a screen for mutants exhibiting hypersensitivity to Hsp90 inhibition via geldanamycin, with aberrant filamentation in the presence of geldanamycin or RPMI (17).

10.1128/mbio.03873-21.1 FIG S1 A strain with decreased *STT4* mRNA levels undergoes invasive filamentous growth. (A) RT-PCR was carried out on the indicated strains (wild type, PY4861; *stt4*Δ/*stt4*-*DAmP1-3*, PY4339-4341) using CaSTT4TM1 and CaSTT4TM2 primer pairs. Fragments migrated at the expected sizes. Values are the means of two determinations with two primer pairs normalized to *ACT1*. (B) Strains were incubated in serum at 37°C for 90 min, and images were acquired. (C) Percentage of filamentous cells (left) (350 cells per strain) and hyphal filament lengths (right) (60 cells per strain) were determined, with error bars indicating SD. (D) Strains were incubated for 4 days at 30°C on agar serum plates, and images were acquired. Download FIG S1, PDF file, 2.7 MB. Copyright © 2022 Garcia-Rodas et al. 2022 Garcia-Rodas et al. https://creativecommons.org/licenses/by/4.0/ This content is distributed under the terms of the Creative Commons Attribution 4.0 International license.

To determine if plasma membrane PI(4)P is essential, we attempted to delete this PI-4-kinase, as well as the two other putative components of the Stt4 complex, *EFR3* and *YPP1*. To generate an *stt4* deletion mutant, we removed the remaining *STT4* copy in the DAmP mutant, by taking advantage of the 3′ *SAT1* marker to target homologous recombination. Southern blotting, as well as PCR of genomic DNA (gDNA), confirmed the absence of *STT4* in this homozygous deletion strain (Fig. S2A and B). In addition, we were able to generate homozygous *EFR3* and *YPP1* deletion mutants, which were verified by PCR (Fig. S3A). Reverse transcription-PCR (RT-PCR) revealed the complete absence of *STT4* mRNA transcript, as well as that of *EFR3* and *YPP1* in the respective mutants (Fig. S2C and S3B). Interestingly, the levels of mRNA transcripts of other PI- and PIP-kinases and phosphatase, including *PIK1*, *MSS4*, and *SAC1*, were unaffected in the *stt4* deletion mutant (Fig. S2C). These strains all grew with doubling times that were indistinguishable from that of the wild-type strain (81 ± 6 min for the wild-type strain compared to 88 ± 7 min for the *stt4*Δ/*stt4*Δ strain, 86 ± 5 min for the *stt4*Δ/*stt4*Δ+*STT4* strain, 86 ± 2 min for the *efr3*Δ/*efr3*Δ strain, and 89 ± 1 min for the *ypp1*Δ/*ypp1*Δ strain), indicating that the PI-4-kinase complex is not necessary for viability or yeast-phase growth in C. albicans.

10.1128/mbio.03873-21.2 FIG S2 Molecular analyses of *stt4* deletion mutants. (A) Southern blot analysis of *stt4* mutants. Schematic representation of chromosomal restriction sites and probes (*STT4* probe, CaStt4p5199 and CaStt4m5543; *URA3* probe, CaUra3pXhoI and CaUra3m81) (left) and Southern blot using *STT4* and *URA3* probes (size in kilobases) of the indicated strains (wild-type, PY4861; *stt4*Δ/*stt4*-DaMP, PY4339; *stt4*Δ/Δ, PY4377 and PY4378) (right) (B) PCR analyses of *stt4* mutants. Schematic representation of *STT4* with primers used for strain verification indicated (top) and PCR analyses of indicated strains (wild-type, PY4861; *stt4*Δ/Δ-1, PY5111; *stt4*Δ/Δ::*STT4*, PY5131; *stt4*Δ/Δ-2, PY4414; *stt4*Δ/Δ *RPS1*::*STT4*, PY4433) (bottom). Primers: 1, CaStt4p4100; 2, CaStt4m4255; 3, CaStt4p5519; 4, CaStt4m6600NotI; 5, CaStt4pup325; 6, CaHIS1pStop1008; 7, CaHIS1mup152; 8, CaHIS1p214; 9, CaHIS1m836; 10, CaARG4m537; 11, CaStt4p4884; 12, CaStt4m5857. The star indicates the position of mutation encoding G1810D. (C) *PIK1*, *MSS4*, and *SAC1* mRNA transcript levels are not altered in the *stt4* mutant. RT-PCR was carried out on the indicated strains (wild type, PY4861; *stt4*Δ/Δ+*STT4*, PY5131; *stt4*Δ/Δ, PY5111) with primers for indicated gene amplifications. Fragments migrated at the expected sizes, and *ACT1* controls revealed similar amounts of cDNA in each strain. Download FIG S2, PDF file, 1.2 MB. Copyright © 2022 Garcia-Rodas et al. 2022 Garcia-Rodas et al. https://creativecommons.org/licenses/by/4.0/ This content is distributed under the terms of the Creative Commons Attribution 4.0 International license.

10.1128/mbio.03873-21.3 FIG S3 Molecular analyses of *efr3* and *ypp1* deletion mutants. (A) PCR analyses of *efr3* and *ypp1* mutants. Schematic representation of *EFR3* and *YPP1* with primers used for strain verification indicated (top) and PCR analyses of indicated strains (wild-type, PY4861; *efr3*Δ/Δ, PY4036; *efr3*Δ/Δ+*EFR3*, PY4039; *ypp1*Δ/Δ, PY4033; *ypp1*Δ/Δ+*YPP1*, PY4040), bottom. Primers: 1, CaYpp1pup167; 2, CaHIS1pStop1008; 3, CaYpp1m3313; 4, CaHIS1mup152; 5, CaURA3p751; 6, CaURA3mup270; 7, CaYpp1m87; 8, CaRPS1p; 9, CaEfr3pup100; 10, CaEfr3m3118; 11, CaEfr3p2692; 12, CaEfr3m127. (B) *efr3*, *ypp1*, and *stt4* mutants are lacking only the respective mRNA transcripts. RT-PCR was carried out on indicated strains (as above, as well as *stt4*Δ/Δ, [PY5111] and *stt4*Δ/Δ+*STT4* [PY5131]) with primers for indicated gene amplifications. Fragments migrated at the expected sizes, and *ACT1* controls revealed similar amounts of cDNA in each strain. Download FIG S3, PDF file, 1.2 MB. Copyright © 2022 Garcia-Rodas et al. 2022 Garcia-Rodas et al. https://creativecommons.org/licenses/by/4.0/ This content is distributed under the terms of the Creative Commons Attribution 4.0 International license.

**FIG 1:**
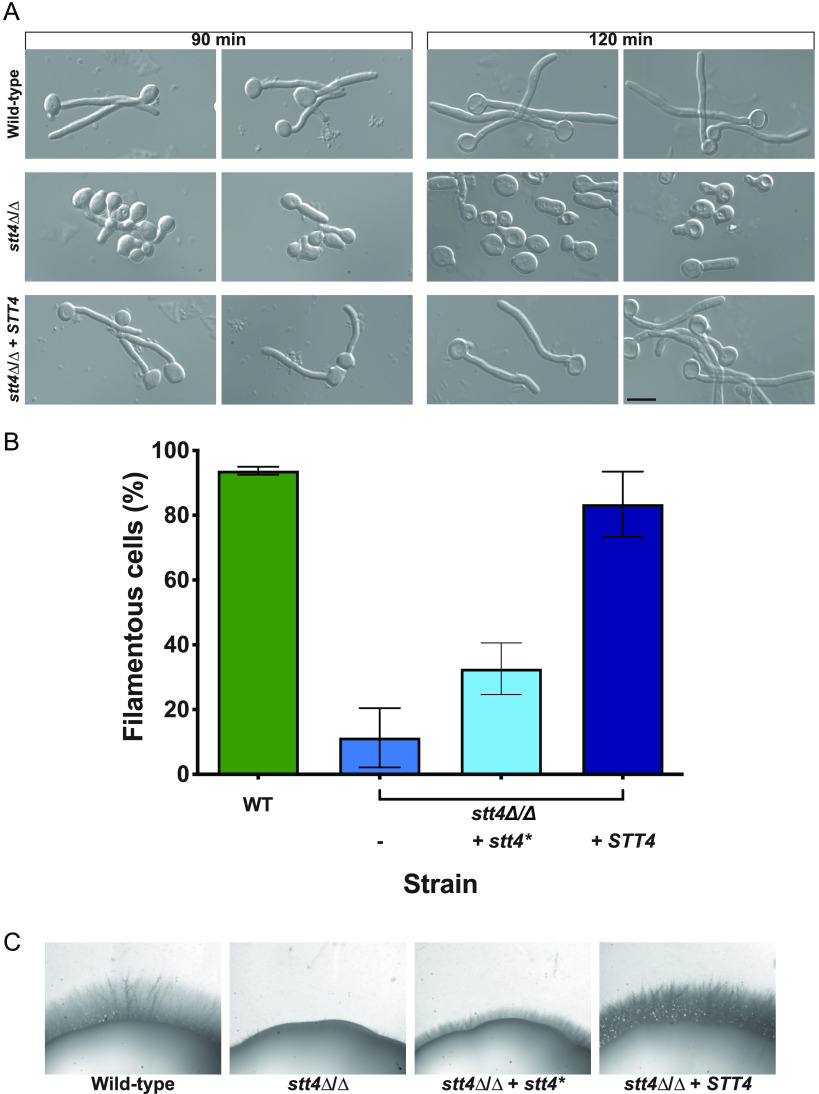
The PI-4-kinase Stt4 is required for filamentous growth. (A) Strains (wild type, PY4861; *stt4*Δ/Δ, PY5111; *stt4*Δ/Δ+*STT4*, PY5131) were incubated with serum at 37°C for 90 and 120 min. Bar, 5 μm. (B) Percentages of filamentous cells were determined from three independent experiments (*n* ≥ 120 in each) with the abovementioned strains in addition to PY5757, an *stt4*Δ/Δ+stt4* strain encoding Stt4[G1810D]. Cells were considered filamentous if germ tubes were twice length of the mother cell or longer. Error bars indicate standard deviations (SD). (C) Stt4 is required for invasive filamentous growth. Strains were incubated for 4 days at 30°C on serum agar plates. Similar results were observed in three independent experiments.

**FIG 2:**
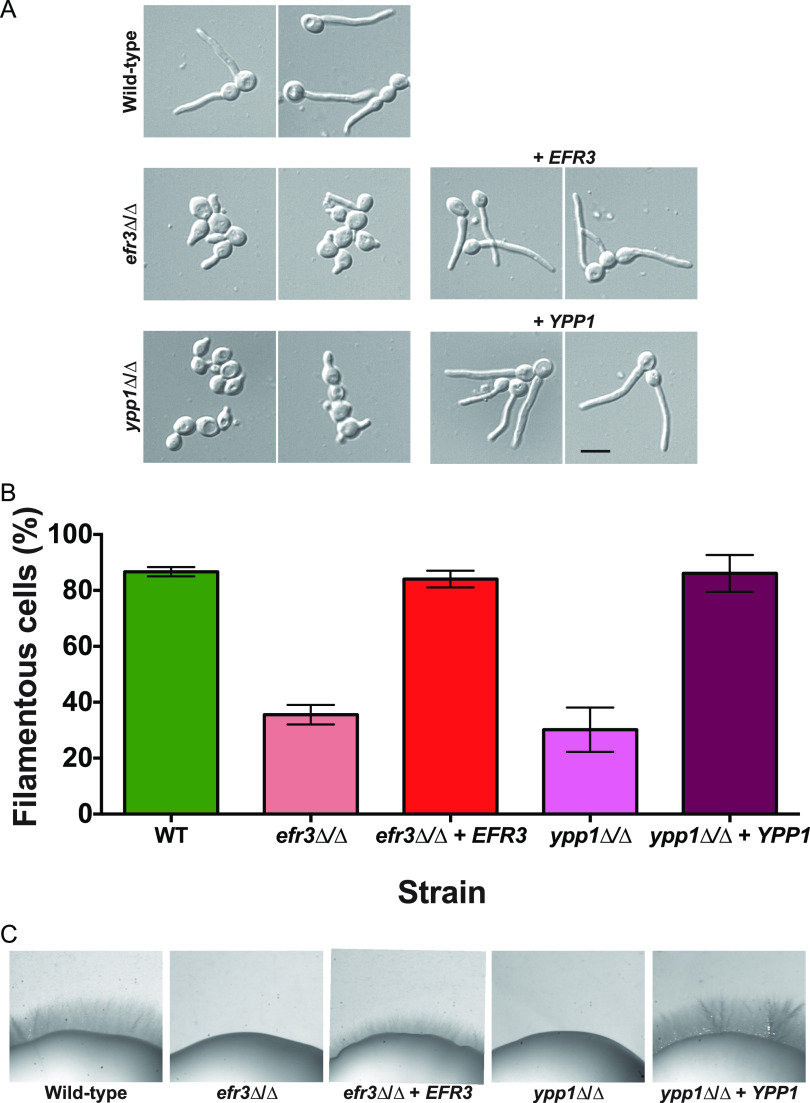
Efr3 and Ypp1 are required for filamentous growth. (A) Indicated strains (wild type, PY4861; *efr3*Δ/Δ, PY4036; *efr3*Δ/Δ+*EFR3*, PY4039; *ypp1*Δ/Δ, PY4033; *ypp1*Δ/Δ+*YPP1*, PY4040) were induced with serum at 37°C for 90 min. Bar, 5 μm. (B) The percentage of filamentous cells was determined from three independent experiments (*n* ≥ 120 in each). Error bars indicate SD. (C) Efr3 and Ypp1 are required for invasive filamentous growth. Strains were incubated for 4 days at 30°C on serum agar plates. Similar results were observed in three independent experiments.

**FIG 3:**
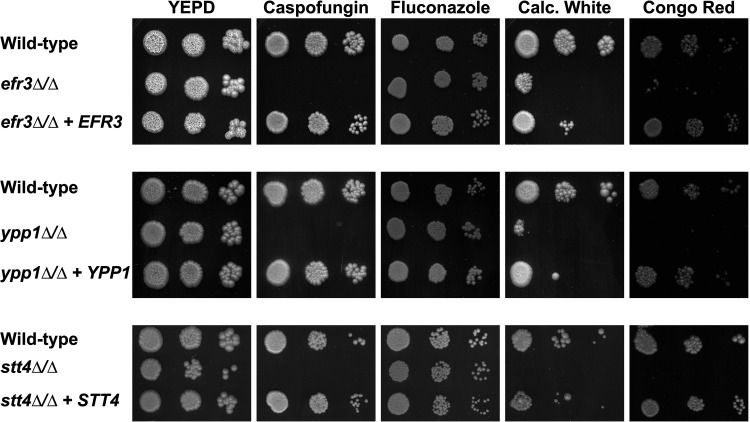
Plasma membrane PI(4)P is important for cell wall integrity. The indicated strains (wild type, PY4861; *efr3*Δ/Δ, PY4036; *efr3*Δ/Δ+*EFR3*, PY4039; *ypp1*Δ/Δ, PY4033; *ypp1*Δ/Δ+*YPP1*, PY4040; *stt4*Δ/Δ, PY5111; *stt4*Δ/Δ+*STT4*, PY5131) were incubated on YEPD with or without caspofungin (125 ng/mL), fluconazole (10 μg/mL), calcofluor white (25 μg/mL), or Congo red (400 μg/mL) for 3 days at 30°C. Similar results were observed in two independent experiments.

In the presence of serum, however, we observed a striking filamentous growth defect (Fig. 1 and 2) in the *efr3*, *ypp1*, and *stt4* deletion mutants. The mutants appeared to form short germ tubes but were unable to form longer hyphal filaments. Similarly, all three strains were completely defective in invasive growth on agar medium containing fetal calf serum (Fig. 1C and 2C). These defects were complemented by the reintroduction of a copy of the respective genes, which was confirmed by PCR of gDNA and RT-PCR of mRNA transcripts (Fig. S2B and C and S3A and B). An *stt4* allele that is a hypomorph with reduced *in vivo* lipid kinase activity (25) was identified in S. cerevisiae in a genetic screen for aminophospholipid transport mutants (7). Hence, we also examined whether such a mutant, in which a highly conserved amino acid in the catalytic domain, Gly1782, was changed to Asp (in C. albicans G1810D), was critical for hyphal growth. Figure 1B shows that a strain expressing as a sole copy this mutated version of *STT4* exhibited defects in filamentous growth that were intermediate between the *stt4* deletion mutant and the complemented strain. Furthermore, compared to the wild-type and complemented strains, the *efr3*, *ypp1*, and *stt4* deletion mutants did not grow in the presence of the cell wall perturbants, including the antifungal drug caspofungin, calcofluor white, and Congo red (Fig. 3). A lower concentration of caspofungin (50 ng/mL compared to 125 ng/mL) revealed that the *stt4* deletion mutant exhibits the greatest sensitivity to this antifungal drug, with the *ypp1* deletion mutant exhibiting intermediate sensitivity and the *efr3* deletion mutant having the lowest sensitivity (Fig. S4A). These mutants were, however, not temperature sensitive and grew similarly to wild-type controls at 30°C and 37°C (Fig. S4B). In contrast to what has been previously reported (26), they did not exhibit an increased sensitivity to fluconazole.

10.1128/mbio.03873-21.4 FIG S4 *efr3*, *ypp1*, and *stt4* deletion mutants are increasingly sensitive to caspofungin and grow similarly to the wild-type strain at 30°C and 37°C. (A) Strains (wild type, PY4861; *efr3*Δ/Δ, PY4036; *efr3*Δ/Δ+*EFR3*, PY4039; *ypp1*Δ/Δ, PY4033; *ypp1*Δ/Δ+*YPP1*, PY4040; *stt4*Δ/Δ, PY5040; *stt4*Δ/Δ+*STT4*, PY5119) were spotted on YEPD agar containing caspofungin at the indicated concentration and incubated for 2 days at 30°C. (B) Indicated strains (wild type, PY4861; *efr3*Δ/Δ, PY4036; *efr3*Δ/Δ+*EFR3*, PY4039; *ypp1*Δ/Δ, PY4033; *ypp1*Δ/Δ+*YPP1*, PY4040; *stt4*Δ/Δ, PY5040; *stt4*Δ/Δ+stt4* encoding Stt4[G1810D], PY5757; *stt4*Δ/Δ+*STT4*, PY5119) were spotted on YEPD agar and incubated for 2 days at the indicated temperature. Download FIG S4, PDF file, 1.6 MB. Copyright © 2022 Garcia-Rodas et al. 2022 Garcia-Rodas et al. https://creativecommons.org/licenses/by/4.0/ This content is distributed under the terms of the Creative Commons Attribution 4.0 International license.

**FIG 4:**
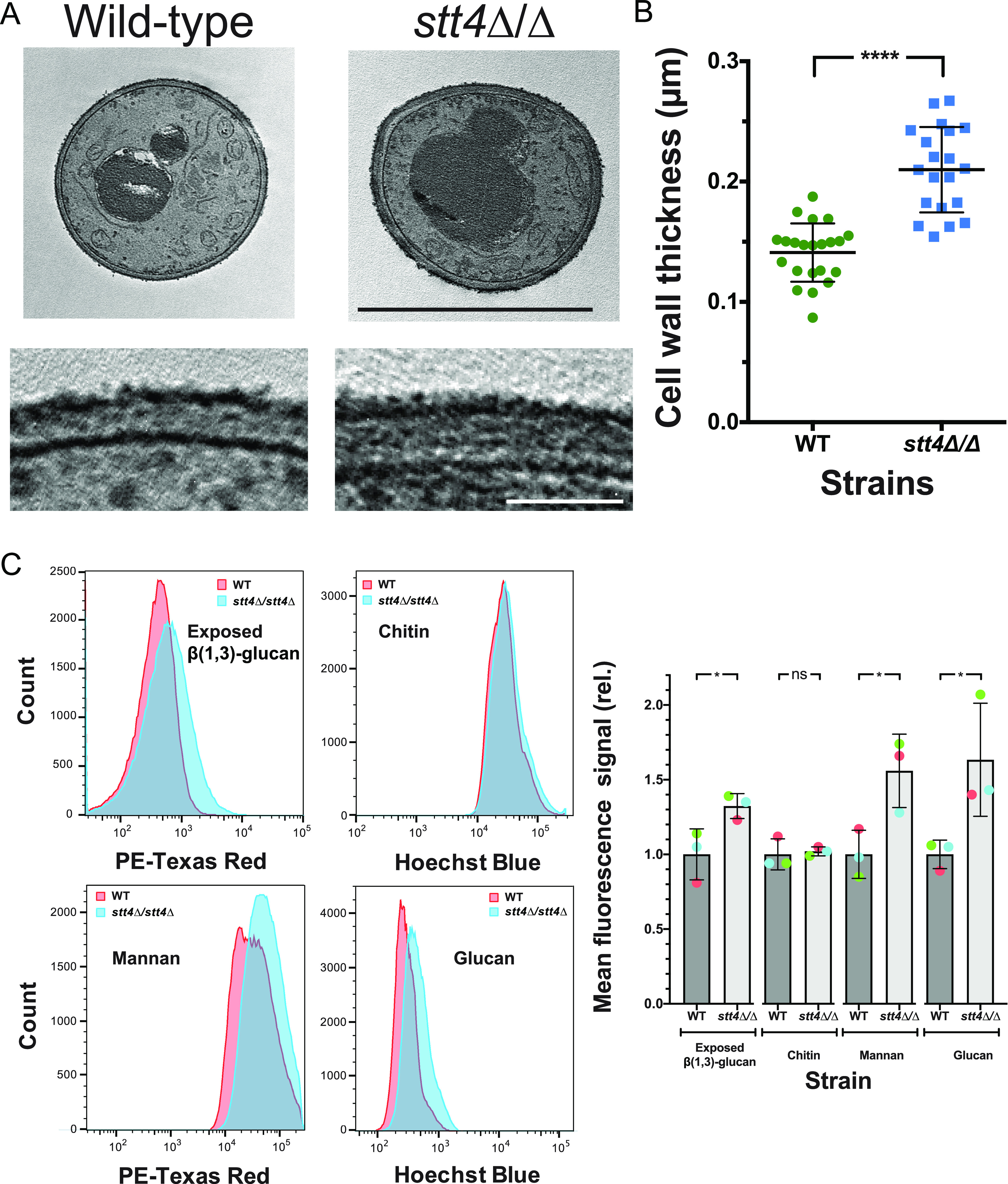
The *stt4* deletion mutant has a thicker cell wall with increased mannan, glucan, and exposed β(1,3)-glucan. (A) Transmission electron micrographs of the indicated strains (wild type, PY4861; *stt4*Δ/Δ, PY5111) (top), with higher magnification of the cell wall (bottom). Bars, 5 μm (top) and 1 μm (bottom). (B) Quantitation of cell wall thickness from electron micrographs. (C) The *stt4* mutant has increased exposure of β(1,3)-glucan together with increased levels of mannan and glucan. Flow cytometry analyses of cells (wild type, PY4861; *stt4*Δ/Δ, PY5111) labeled with anti-β(1,3)-glucan antibodies and a fluorescently labeled secondary antibody, calcofluor white, fluorescently labeled concanavalin A, and aniline blue. Flow cytometry profiles from one biological replicate (10^5^ gated events; left) and means from three biological replicates normalized to each wild-type mean (right). Error bars indicate standard deviations. * *P* < 0.05; ****, *P* < 0.0001; ns, not significant.

Given the effects of cell wall perturbants, we examined the cell wall thickness and composition in the *stt4* homozygous deletion mutant. Figure 4A and B show that, in this PI-4-kinase deletion mutant, the cell wall was on average 50% thicker than that in wild-type cells. Flow cytometry was used to quantitate the exposed β(1,3)-glucan, as well as the total chitin, mannan, and glucan. A significant increase in exposed β(1,3)-glucan, mannan, and glucan content was observed in the *stt4* deletion mutant compared to the wild-type strain (Fig. 4C). On average *stt4* mutant cells had a 30% increase in exposed β(1,3)-glucan and a 60% increase in mannan and glucan content. This cell wall integrity defect and increase in cell surface exposure of β(1,3)-glucan are reminiscent of that observed in the *cho1*Δ/*cho1*Δ phosphatidylserine synthase mutant (27–30). Together, our results show that the PI-4-kinase complex is critical for filamentous growth and cell wall integrity in C. albicans, specifically masking cell wall β(1,3)-glucan.

### The Stt4 PI-4-kinase complex is specifically required for plasma membrane PI(4)P.

**FIG 5:**
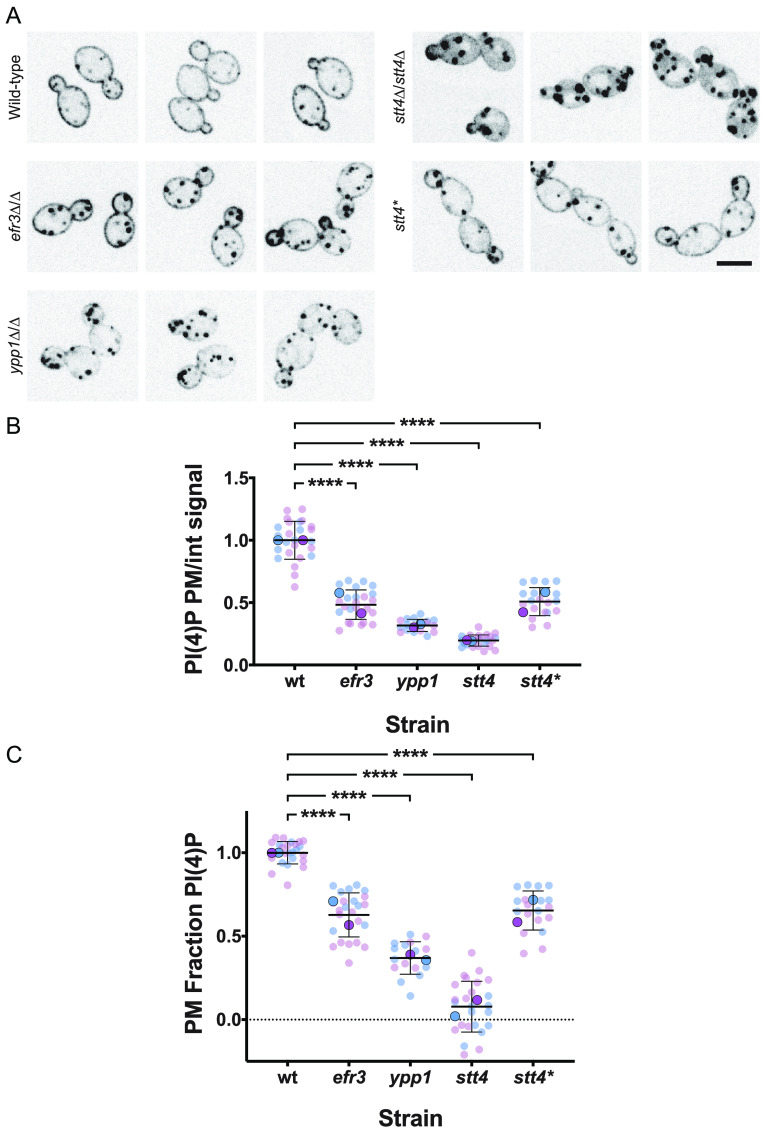
Efr3, Ypp1, and Stt4 are critical for plasma membrane PI(4)P. (A) Strains expressing the plasma membrane PI(4)P reporter, GFP-Osh2^PH^-Osh2^PH^-GFP (wild type, PY2626; *efr3*Δ/Δ, PY4947; *ypp1*Δ/Δ, PY3950; *stt4*Δ/Δ, PY5169; *stt4*Δ/Δ+stt4*, PY5838) were imaged, and central z-sections of representative cells are shown with an inverted lookup table (LUT). Bar, 5 μm. (B and C) Quantitation of plasma membrane and internal signals reveals little to no plasma membrane PI(4)P in the *stt4* mutant. The ratio of plasma membrane to internal signal (normalized to the wild type) and the relative plasma membrane signal (normalized plasma membrane/total signal) is shown. Quantitation of plasma membrane and internal signals was carried out excluding Golgi cisternae. For the wild type, the mean ratio of plasma membrane to internal signal was 3.8 and the ratio of plasma membrane to total signal was 0.8. We were able to detect ∼1.5% of wild-type plasma membrane PI(4)P levels. Smaller symbols are values from two experiments (6 to 15 cells per experiment), and larger symbols are means for each experiment, with bars indicating overall means and standard deviations. ****, *P* < 0.0001.

The defect in filamentous growth in mutants for all three components of the PI-4-kinase complex suggested that plasma membrane PI(4)P is critical for this process. Therefore, we examined the *in vivo* PI(4)P levels using a fluorescent reporter that binds preferentially this acidic phospholipid at the plasma membrane (13). This reporter can also bind PI(4)P at the Golgi apparatus, and we have previously shown that, upon a reduction in plasma membrane PI(4)P, the reporter relocalizes to the Golgi apparatus (13). In wild-type cells, we observed this green fluorescent protein (GFP)-PH^OSH2^-PH^OSH2^-GFP reporter localize predominantly at the plasma membrane, yet in *efr3*, *ypp1*, and *stt4* mutants, there was a decrease in plasma membrane PI(4)P, with a concomitant increase in intracellular Golgi PI(4)P signal (Fig. 5A). Quantification of the reporter fluorescence from these central z-sections, using the Matlab program Hyphal-Polarity (14), indicated that the ratio of mean plasma membrane signal to mean internal signal decreased progressively in the *efr3*, *ypp1*, and *stt4* mutants, resulting from the decrease in plasma membrane PI(4)P together with the overall increase in internal PI(4)P levels (Fig. 5B). Closer examination of *stt4* mutant cells revealed little to no plasma membrane PI(4)P (Fig. 5A), and this was confirmed by quantification of the normalized fraction of total signal at the plasma membrane, i.e., the plasma membrane signal excluding the Golgi cisternae divided by the total cell signal excluding the Golgi cisternae (Fig. 5C). The average normalized plasma membrane PI(4)P in *stt4* mutants was very close to 0 (0.078 ± 0.016), with 60% of cells (*n* = 200) having undetectable plasma membrane PI(4)P. Together, these results indicate that C. albicans cells with undetectable plasma membrane PI(4)P are viable and suggest that this plasma membrane acidic phospholipid is specifically critical for filamentous growth.

**FIG 6:**
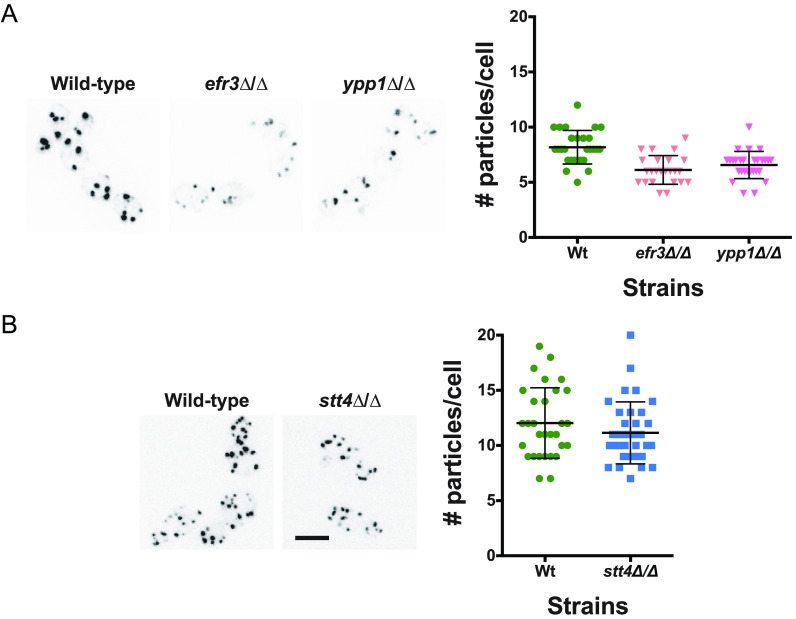
The number of Golgi cisternae is not affected by a decrease in plasma membrane PI(4)P. (A and B) Strains expressing Golgi PI(4)P reporter, FAPP1-GFP (wild type, PY2578; *efr3*Δ/Δ, PY3933; *ypp1*Δ/Δ, PY3951; *stt4*Δ/Δ, PY5552) were imaged, and maximum projections of representative cells are shown with an inverted LUT (left). Quantitation of the number of Golgi cisternae per cell (right) in the indicated strains (24 to 34 cells per strain). Bar, 5 μm.

**FIG 7:**
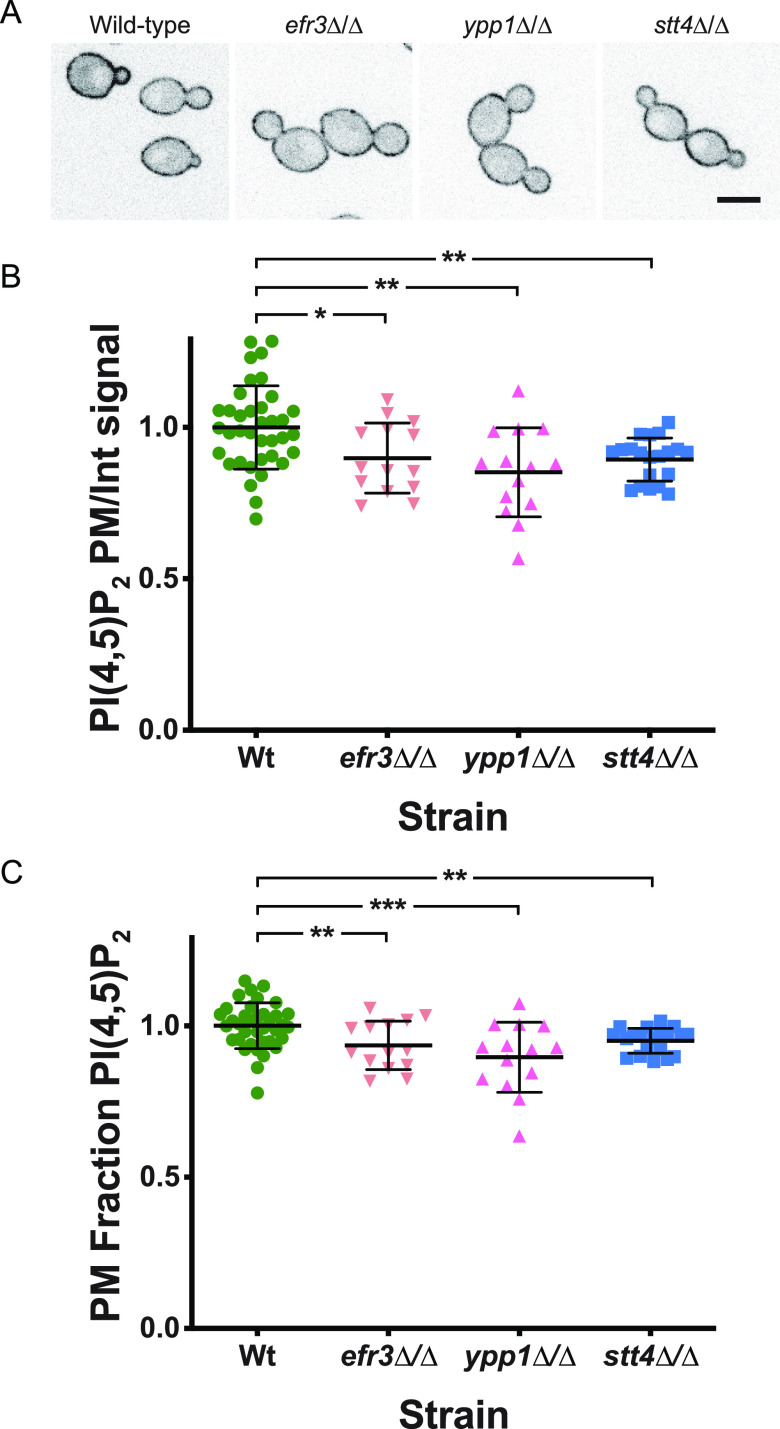
Plasma membrane PI(4,5)P_2_ is not substantially affected by a decrease in PI(4)P. (A) Strains expressing the PI(4,5)P_2_ reporter GFP-PH^Plcδ^-PH^Plcδ^-GFP (wild type, PY1206; *efr3*Δ/Δ, PY3935; *ypp1*Δ/Δ, PY3958; *stt4*Δ/Δ, PY555) were imaged, and central z-sections of representative cells are shown with an inverted LUT. Bar, 5 μm. (B and C) Quantitation of plasma membrane and internal signals reveals that plasma membrane PI(4,5)P_2_ is largely unaffected in the absence of Efr3, Ypp1, and Stt4. The ratio of plasma membrane to internal signal and the relative plasma membrane signal were determined as for Fig. 5B and C (15 to 20 cells; 2 experiments for the wild type [WT]). For the wild type, the mean ratio of plasma membrane to internal signal was 2.8 and the ratio of plasma membrane to total signal was 0.7. *, *P* < 0.02; **, *P* < 0.01; ***, *P* < 0.0005.

Previously, we showed that a reduction in Golgi PI(4)P results in Golgi cisternae proliferation (13). Hence, we examined whether the reduction of plasma membrane PI(4)P observed in *efr3*, *ypp1*, and *stt4* deletion mutants affected the Golgi cisternae. While there was a small decrease (20 to 25%) in the number of Golgi cisternae per cell in *efr3* and *ypp1* mutants compared to the wild-type (Fig. 6A), there was no difference in the number of Golgi cisternae per cell in the *stt4* mutant (Fig. 6B). These results further confirm that Golgi apparatus and plasma membrane PI(4)P pools are functionally distinct. Our previous analyses of a *STT4* repressible strain revealed that upon a 10-fold repression of *STT4* transcript, there was a reduction of plasma membrane PI(4,5)P_2_ (14). As a result, we examined plasma membrane PI(4,5)P_2_ levels in the *efr3*, *ypp1*, and *stt4* deletion mutants. Figure 7 shows that this phosphatidylinositol phosphate was observed at the plasma membrane in all three mutants. Compared to the wild-type strain, there was a reduction of 10 to 15% in the plasma membrane PI(4,5)P_2_ levels to internal ratios in the *efr3*, *ypp1*, and *stt4* mutants. Comparison of the fraction of plasma membrane PI(4,5)P_2_ signal revealed a small (10% or less) reduction in the *stt4* mutants with respect to the wild-type strain, similar to our previous observations with a repressible strain (14). Together, these results demonstrate that the dramatic reduction of plasma membrane PI(4)P does not alter Golgi PI(4)P, nor does it substantially alter plasma membrane PI(4,5)P_2_.

**FIG 8:**
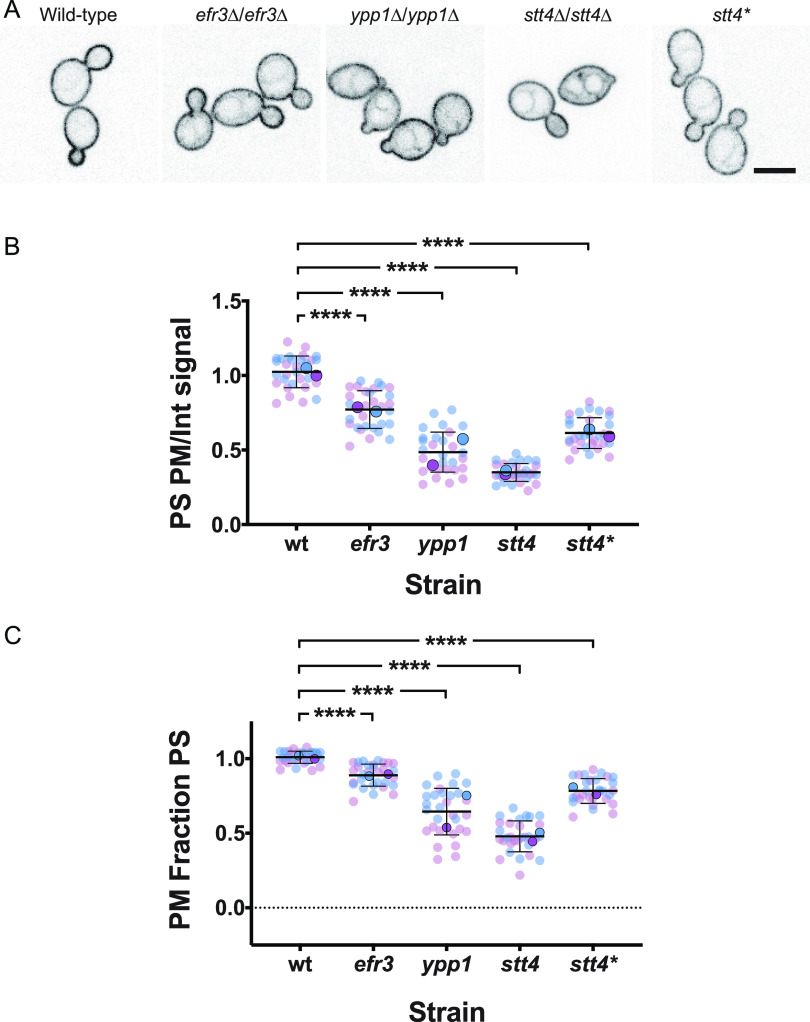
A reduction in plasma membrane PI(4)P results in an increase in PS at the ER. (A) Strains expressing PS reporter GFP-LactC2 (wild type, PY3239; *efr3*Δ/Δ, PY4124; *ypp1*Δ/Δ, PY4131; *stt4*Δ/Δ, PY5174; *stt4*Δ/Δ+stt4*, PY5903) were imaged, and central z-sections of representative cells are shown with an inverted LUT. Bar, 5 μm. (B and C) Quantitation of plasma membrane and internal signals reveals a progressive decrease in plasma membrane PS in *efr3*, *ypp1*, and *stt4* strains. The ratio of plasma membrane to internal signal and the relative plasma membrane signal were determined as for Fig. 5B and C. For the wild type, the mean ratio of plasma membrane to internal signal was 4.5 and the ratio of plasma membrane to total signal was 0.8. Smaller symbols are values from two experiments (15 cells each); larger symbols are means from each experiment, with bars indicating overall means and standard deviations. ****, *P* < 0.0001.

**FIG 9:**
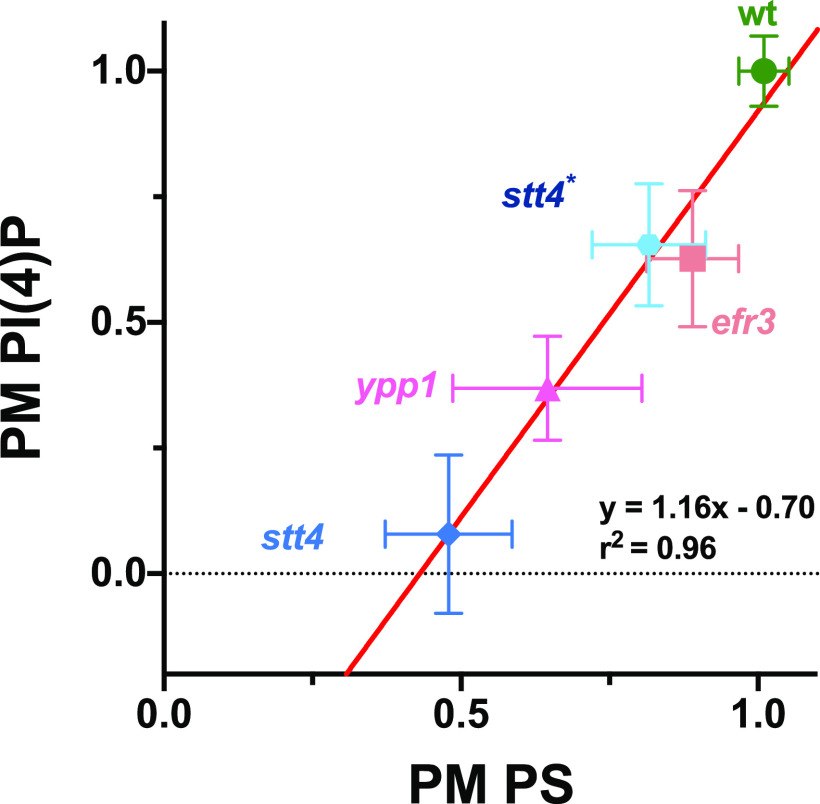
Plasma membrane PS is proportional to PI(4)P levels. Average levels of plasma membrane PS and PI(4)P in indicated strains were normalized to 1 in the wild type. Linear curve fit: *y*=1.316*x*−0.70; *r*^2^ = 0.96. Bars show standard deviations for 16 to 40 cells for each determination.

Oxysterol-binding proteins, such as Osh6 and Osh7 in S. cerevisiae, are lipid transfer proteins that transfer phosphatidylserine (PS) from the ER to the plasma membrane concomitant with transfer of PI(4)P from the plasma membrane to the ER, where it is hydrolyzed by Sac1 (31–33). In C. albicans, as in S. cerevisiae, Sac1, which localizes to the ER and Golgi apparatus, is critical for regulating plasma membrane PI(4)P levels (13, 34, 35). Given that in the *efr3*, *ypp1*, and *stt4* deletion mutants there was a reduction in PI(4)P levels, we examined whether PS plasma membrane levels were affected using a fluorescent reporter that preferentially binds this acidic phospholipid (36). In wild-type cells, we observed this LactC2-GFP reporter localized predominantly at the plasma membrane, with little to no internal signal observed (Fig. 8A). In contrast, in the *efr3*, *ypp1* and *stt4* mutants, a perinuclear signal was observed, characteristic of the ER, yet plasma membrane PS was still apparent in each of these mutants (Fig. 8A). Quantification of signals from central z-sections using the Matlab program Hyphal-Polarity (14) confirmed that the ratio of mean plasma membrane signal to mean internal signal decreased progressively in the *efr3*, *ypp1*, and *stt4* mutants, resulting in part from a progressive increase in internal PS signal (Fig. 8B). Similarly, the mean plasma membrane PS fraction also decreased progressively in the *efr3*, *ypp1*, and *stt4* mutants, with the *stt4* deletion mutant exhibiting an approximately 50% reduction compared to wild-type cells. The *stt4* hypomorph, Stt4[G1810D], had plasma membrane PS levels intermediate between those of the *efr3* and *ypp1* mutants. Together, these results suggest that sufficient PI(4)P is critical for the transport of PS from the ER to the plasma membrane. We next examined if there was a correlation between the plasma membrane PI(4)P and PS levels. Figure 9 shows that there is a direct correlation between the levels of these two lipids in the different deletion mutants and the wild-type strain. Plasma membrane lipid levels in the Stt4[G1810D] mutant were also consistent with such a correlation. Note that while we were unable to detect plasma membrane PI(4)P in the *stt4* deletion strain, approximately 50% of the plasma membrane PS was detectable in this mutant. Furthermore, plasma membrane PS levels in the *efr3* mutant were not dramatically different from that of the wild-type cells. Together, our results suggest that the filamentation and cell wall integrity defects observed in the three Stt4 complex mutants are likely to be due to lack of plasma membrane PI(4)P and not PS.

### Stt4 PI-4-kinase complex localizes to cortical patches.

**FIG 10:**
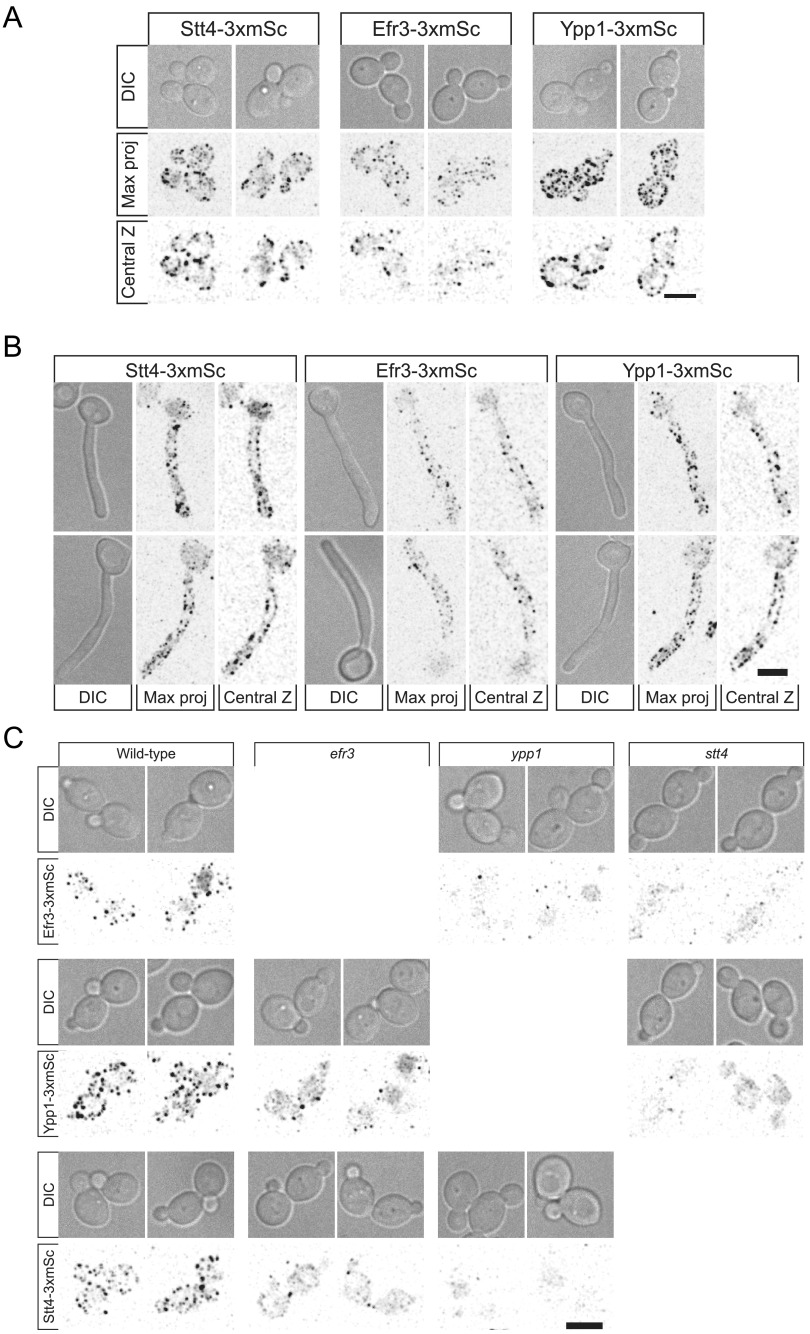
Efr3, Ypp1, and Stt4 localize as cortical patches, with Ypp1 and Stt4 being critical for each other’s localization. (A and B) Strains expressing indicated 3xmScarlet fusions (Stt4-3xmSc, PY6193; Efr3-3xmSc, PY6197; Ypp1-3xmSc, PY6195) were imaged during budding (A) and hyphal (B) growth. Differential interference contrast (DIC) images, central z-sections, and maximum projections of 17 0.5-μm z-sections are shown. (C) Strains (WT, PY6197, PY6195, and PY6193; *efr3*, PY6136 and PY6142; *ypp1*, PY6138 and PY6144; *stt4*, PY6140 and PY6134) expressing respective 3xmScarlet fusions were imaged during budding growth, and maximum projections of 17 0.5-μm z-sections are shown. Bars, 5 μm.

To determine the distribution of Efr1, Ypp1, and Stt4, we generated 3×-mScarlet fusions by tagging the chromosomal copy of the respective genes. These fusions were functional in that as a sole copy, they complemented the cell wall integrity defect of the respective mutants (Fig. S5). Despite the low abundance of these Stt4 PI-4-kinase complex subunits, we observed patches around the cortex of the mother cell and buds (Fig. 10A), which were also visible along the cortex of the germ tubes with reduced signals in the mother cell (Fig. 10B). In S. cerevisiae, *YPP1* and *EFR3* are critical for Stt4 membrane localization. Here, we observed that while Stt4 localization to the cortex is dependent on Ypp1 in C. albicans, in the *efr3* mutant there were still some cells with cortex-localized Stt4 (Fig. 10C). Efr3 cortex localization depended upon Ypp1 and Stt4, with loss of cortex signal observed in either mutant. In contrast, there were some cells with *YPP1* cortex signal in the *efr3* mutant but not in the *stt4* mutant. In this *efr3* mutant, 40 to 50% of cells exhibited punctate localization of Stt4 and Ypp1. In the *ypp1* mutant, we did not observe cells with either Stt4 or Efr3 localized. Finally, in the *stt4* mutant, only ∼10% of cells exhibited punctate localization of Ypp1. RT-PCR revealed that the mScarlet transcript levels in all mutants were similar to that of the wild type (Fig. S6). As these results suggested that Ypp1 and Stt4 were the most critical components of this PI-4-kinase complex, we examined whether these proteins colocalized to the same cortical patches. Figure S7 shows that, in a strain expressing Ypp1-mTurquoise and Stt4-3xmScarlet, there was only limited colocalization of these two proteins, suggesting that, if they form a complex, it is likely to be transient.

10.1128/mbio.03873-21.5 FIG S5 The Efr3, Ypp1, and Stt4 3× mScarlet fusions are functional. The indicated strains (*efr3*Δ/*EFR3*-3xmSc, PY5599; *efr3*Δ/Δ, PY4036; wild-type, PY4861; *ypp1*Δ/*YPP1*-3xmSc, PY5574; *ypp1*Δ/Δ, PY4033; *stt4*Δ/*STT4*-3xmSc, 5601; *stt4*Δ/*stt4*Δ, PY5111) were spotted on YEPD containing calcofluor white (25 μg/mL) and incubated for 2 days at 30°C. Download FIG S5, PDF file, 0.6 MB. Copyright © 2022 Garcia-Rodas et al. 2022 Garcia-Rodas et al. https://creativecommons.org/licenses/by/4.0/ This content is distributed under the terms of the Creative Commons Attribution 4.0 International license.

10.1128/mbio.03873-21.6 FIG S6 Transcript levels of *EFR1*, *YPP1*, and *STT4* mScarlet fusions are not altered in the PI-4-kinase complex mutants. RT-PCR was carried out on the indicated strains (WT, PY6195 Ypp1-3xmSc, PY6197 Efr3-3xmSc, PY6193 Stt4-3xmSc; *stt4* Ypp1-3xmSc, PY6134; *stt4* Efr3-3xmSc, PY6140; *ypp1* Efr3-3xmSc, PY6138; *ypp1* Stt4-3xmSc, PY6144; *efr3* Ypp1-3xmSc, PY6136; *efr3* Stt4-3xmSc; WT, PY4860) with primers (CamSCARLETp, CamSCARLETm, CaACT1p, and CaACT1m) and for indicated gene amplifications. Fragments migrated at the expected sizes, and *ACT1* controls revealed similar amounts of cDNA in each strain. Similar results were observed with two additional mScarlet primer pairs. Download FIG S6, PDF file, 0.1 MB. Copyright © 2022 Garcia-Rodas et al. 2022 Garcia-Rodas et al. https://creativecommons.org/licenses/by/4.0/ This content is distributed under the terms of the Creative Commons Attribution 4.0 International license.

10.1128/mbio.03873-21.7 FIG S7 Ypp1 and Stt4 do not colocalize in cortical patches. A strain expressing Stt4-3xmScarlet (magenta) and Ypp1-mTurquoise (green), PY6201, was imaged during budding growth, and maximum projections of 17 0.5-μm z-sections are shown. Bar, 5 μm. Download FIG S7, PDF file, 0.1 MB. Copyright © 2022 Garcia-Rodas et al. 2022 Garcia-Rodas et al. https://creativecommons.org/licenses/by/4.0/ This content is distributed under the terms of the Creative Commons Attribution 4.0 International license.

### Plasma membrane PI(4)P is critical for virulence.

**FIG 11:**
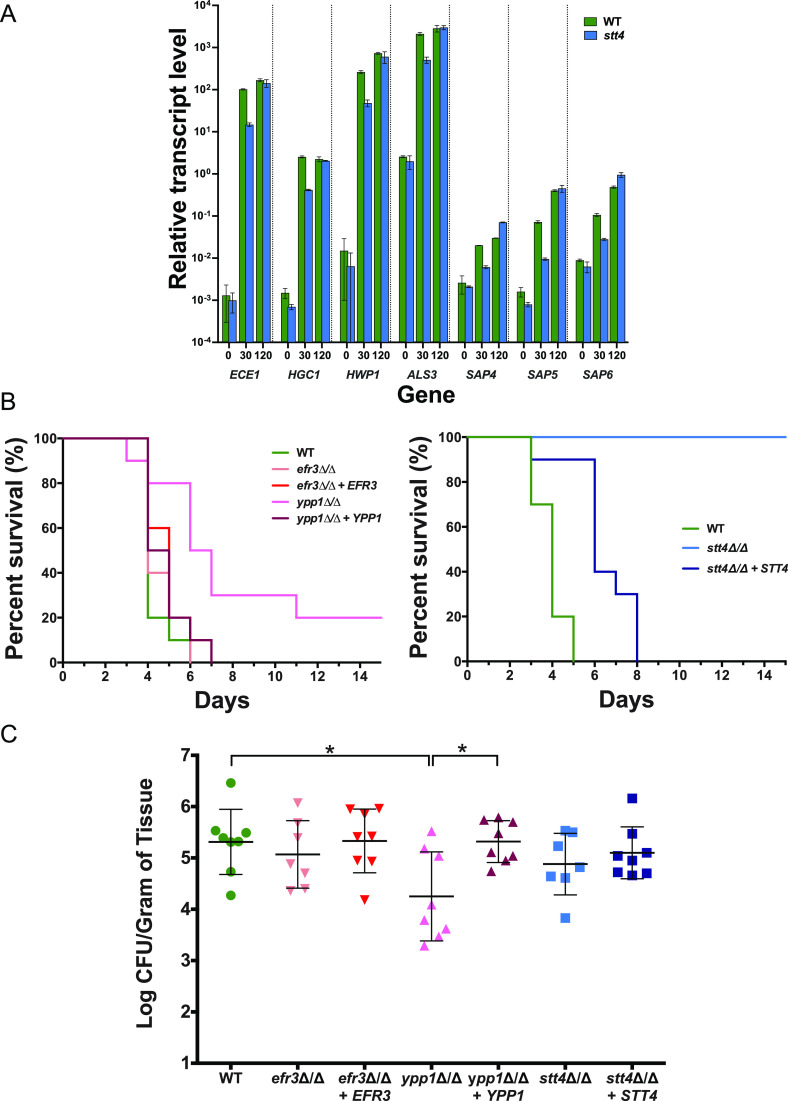
Plasma membrane PI(4)P is specifically required for hematogenously disseminated candidiasis. (A) Hyphal-specific genes are induced in an *stt4* deletion mutant. The transcript level of indicated hyphal specific genes was determined in wild-type (PY4861) and *stt4*Δ/Δ (PY5111) strains at indicated times (in minutes) with serum at 37°C by qRT-PCR and normalized to the levels of TDH3 transcript. Means of 3 determinations from an experiment are shown, with bars indicating standard deviations. Similar results were observed in two additional biological replicates. (B) Stt4 is required for virulence in a murine systemic infection model. Survival of mice (*n* = 10) over time following injection of strains (wild type, PY4861; *efr3*Δ/Δ, PY4036; *efr3*Δ/Δ+*EFR3*, PY4039; *ypp1*Δ/Δ, PY4033; *ypp1*Δ/Δ+*YPP1*, PY4040; *stt4*Δ/Δ, PY5111; *stt4*Δ/Δ+*STT4*, PY5131). Similar results were observed in two independent experiments; the difference between the WT and the *ypp1*Δ/Δ mutant was statistically significant (*P* = 0.002) (left), and that between the WT and the *stt4*Δ/Δ mutant was statistically significant (*P* < 0.0001) (right). (C) Stt4 and Efr3 are not required for virulence in a murine oropharyngeal infection model. CFU per gram of tongue tissue was determined 5 days after oropharyngeal infection (8 mice per strain) with the indicated strains (wild type, PY4861; *efr3*Δ/Δ, PY4036; *efr3*Δ/Δ+*EFR3*, PY4039; *ypp1*Δ/Δ, PY4033; *ypp1*Δ/Δ+*YPP1*, PY4040; *stt4*Δ/Δ, PY4414; *stt4*Δ/Δ+*STT4*, PY4433). *, *P* < 0.05.

To investigate the importance of plasma membrane PI(4)P in virulence, we examined the *efr3*, *ypp1*, and *stt4* mutants in two murine infection models, HDC and oropharyngeal candidiasis (OPC). As C. albicans responds to cues, such as the presence of serum, by the induction of hypha-specific genes (HSGs), many of which are critical for virulence, we initially examined HSG levels after 30 and 120 min incubation with serum. Figure 11A shows that the *stt4* deletion mutant induced a range of HSGs, including *ECE1*, *HGC1*, *HWP1*, *ALS3*, and *SAP4* to -*6*, 10^3^- to 10^5^-fold (excluding *SAP4* to -*6*), similar to the wild-type strain, with only a 5-fold reduction in induced transcript levels observed after 30 min incubation. In the HDC model, 20% and 100% of mice infected with the *ypp1* and *stt4* mutants, respectively, survived 2 weeks after injection, while all of the mice infected with the *efr3* mutant or wild-type strain died within 5 to 6 days (Fig. 11B). The virulence was significantly restored in complemented strains (Fig. 11B). Furthermore, we examined whether the *stt4* deletion mutant could form filaments after long incubation times in serum or in kidney homogenate (37). After 6 h incubation with either serum or kidney homogenate, despite observing elongated cells, the *stt4* mutant did not form hyphal filaments, compared to wild-type and complemented strains (Fig. S8). It is unlikely that the unmasking of cell surface β(1,3)-glucan and these virulence defects are due to altered plasma membrane PS levels, as a *cho1*Δ/*cho1*Δ::*CHO1* strain with >50% reduction in PS levels had no increase in unmasked β(1,3)-glucan and exhibited full virulence in mouse models of systemic and oropharyngeal infection (27–29). In the OPC model, only the *ypp1* mutant was substantially less virulent than the wild-type strain, with the oral fungal burden of the infected mice reduced by ∼25-fold (Fig. 11C). Nonetheless, examination of the histopathology of tongue thin sections revealed that in infection lesions, all strains were able to form filaments (Fig. S9). Together, these data suggest that plasma membrane PI(4)P is required for virulence during hematogenously disseminated candidiasis, with a decrease in virulence observed upon a 60% reduction in plasma membrane PI(4)P and the lack of lethality observed in the absence of this phosphatidylinositol phosphate. Our data also indicate that only a small amount of plasma membrane PI(4)P is required for normal virulence during oropharyngeal candidiasis.

10.1128/mbio.03873-21.8 FIG S8 The *stt4* deletion mutant elongates in response to serum and murine kidney homogenates. Strains (wild-type, PY4861; *stt4*Δ/Δ, PY5111; *stt4*Δ/Δ+*STT4*, PY5131) were incubated with either serum or 0.4 g/mL kidney homogenates for 6 h at 37°C, samples were fixed and stained with calcofluor white, and images (31 0.4-μm z-sections) were acquired. Maximum projections are shown with an inverted LUT. Bar, 5 μm. Download FIG S8, PDF file, 0.3 MB. Copyright © 2022 Garcia-Rodas et al. 2022 Garcia-Rodas et al. https://creativecommons.org/licenses/by/4.0/ This content is distributed under the terms of the Creative Commons Attribution 4.0 International license.

10.1128/mbio.03873-21.9 FIG S9 PI-4-kinase complex mutants can exhibit filamentous growth in a murine OPC model. Histopathology of tongue thin sections from mice infected with indicated strains (see Fig. 11C). Thin sections were stained with periodic acid-Schiff stain. Images of regions of infection are shown to highlight fungal morphology, with enlargements of images in left panels (bar, 50 μm) shown on the right (bar, 100 μm). Note that there were fewer infection sites in mice infected with the *ypp1* mutant, which were also smaller. Download FIG S9, PDF file, 2.8 MB. Copyright © 2022 Garcia-Rodas et al. 2022 Garcia-Rodas et al. https://creativecommons.org/licenses/by/4.0/ This content is distributed under the terms of the Creative Commons Attribution 4.0 International license.

## DISCUSSION

Our results show that plasma membrane PI(4)P is critical for the C. albicans yeast to filamentous growth transition and cell wall integrity. We show that all three members of the Stt4 PI-4-kinase complex are dispensable for viability yet are required for filamentous growth and cell wall integrity. Furthermore, quantitative analyses indicate that these mutants have decreasing levels of plasma membrane PI(4)P, from *efr3* to *ypp1* to *stt4*; a majority of the *stt4* cells lack detectable PI(4)P at the plasma membrane. In addition, an *stt4* hypomorph (25) had plasma membrane PI(4)P levels similar to those of the *efr3* mutant. We observed little to no alteration in Golgi PI(4)P and plasma membrane PI(4,5)P_2_ in these Stt4 PI-4-kinase complex mutants. Consistent with a link between plasma membrane PI(4)P and PS, there is a gradual increase in internal pools of PS in *efr3*, *ypp1*, and *stt4* mutants, yet even in the *stt4* mutant that lacks plasma membrane PI(4)P, PS is still detected at the plasma membrane. All three of these PI-4-kinase complex proteins localize to cortical patches, but only Ypp1 and Stt4 appear to be critical for the complex formation. Furthermore, our results reveal that plasma membrane PI(4)P is important for masking cell surface β(1,3)-glucan but not for induction of a number of hyphal-specific genes. Plasma membrane PI(4)P is critical for pathogenicity during hematogenously disseminated candidiasis but less so for oropharyngeal candidiasis, suggesting that this lipid has different roles in distinct anatomic infection sites, which we attribute, in part, to host immune recognition via unmasked cell surface β(1,3)-glucan.

Using strains in which either the C. albicans PI-4-kinase, i.e., Pik1 at the Golgi apparatus or Stt4 at the plasma membrane, could be repressed, we previously showed that the Golgi PI-4-kinase is strictly required for invasive filamentous growth (13), whereas repression of the plasma membrane PI-4-kinase mutant resulted in cells that can still form short protrusions and invasive filaments (14). This repressible *stt4* mutant had an ∼10-fold reduction in *STT4* transcript levels compared to a wild-type strain, yet PI(4)P was still detectable at the plasma membrane. To address the importance of plasma membrane PI(4)P in C. albicans, we generated homozygous deletion mutants in the PI-4-kinase complex, composed of Efr3, Ypp1, and Stt4. None of these PI-4-kinase components is essential for viability in C. albicans, in contrast to other fungi, specifically S. cerevisiae, where both Pik1 and Stt4 are essential (6, 7, 38, 39). We speculate that the plasma membrane PI(4)P is required to maintain filamentous growth in C. albicans via contributions to cell polarity and membrane traffic. A. nidulans and C. neoformans appear to have only a Stt4 PI-4-kinase, which is essential for viability (4, 5). In addition, although C. albicans *stt4*, *ypp1*, and *efr3* mutants showed increased sensitivity to cell wall perturbants, they did not exhibit a temperature-sensitive growth defect, as was the case in S. cerevisiae, where a temperature-sensitive *stt4* mutant lysed at the nonpermissive temperature (15). Our quantitative analyses revealed that the majority of C. albicans cells lacking Stt4 have undetectable levels of PI(4)P at the plasma membrane, suggesting that this lipid is dispensable for viability. Such C. albicans mutants with little to no plasma membrane PI(4)P are sensitive to cell wall perturbants and have thicker cell walls with increased levels of glucan and mannan, along with an increase in cell surface exposed β(1,3)-glucan, indicating that PI(4)P is critical for maintaining cell wall integrity. We speculate that plasma membrane glucan synthase activity is regulated by PI(4)P, via the Rho1 regulatory subunit (40). This raises the question of how PI(4,5)P_2_ is generated in the *stt4* deletion mutant, and we speculate that PI(4)P from the Golgi apparatus may be phosphorylated by Mss4. This could occur via Mss4 localization to the Golgi apparatus or secretory vesicles, although this kinase has not been detected in these compartments in C. albicans (14). Alternatively, a small amount of Golgi PI(4)P could reach the plasma membrane and be immediately phosphorylated.

Previous studies in S. cerevisiae indicated that the levels of PS and plasma membrane PI(4)P are linked. For example, an S. cerevisiae *stt4* mutant with reduced levels of plasma membrane PI(4)P accumulates PS (7, 25). Furthermore, an S. cerevisiae *sac1* phosphatase mutant that results in a dramatic increase in PI(4)P (34, 35) has decreased PS levels at the plasma membrane, with an increase in intracellular membranes (41). Similarly, in fibroblasts, PI-4-kinase IIIα knockouts have decreased plasma membrane PS (42). The S. cerevisiae oxysterol proteins Osh6 and Osh7 have been shown to exchange PS for PI(4)P *in vitro*, and *in vivo*, a *sac1* mutation resulted in a redistribution of added lyso-PS, which is normally at the plasma membrane, to the ER (32). It was proposed that a PI(4)P gradient from the plasma membrane to the ER drives PS transport via Osh6/7 from the ER to the plasma membrane (32). Given that C. albicans cells are viable with little to no plasma membrane PI(4)P, it is likely that this so-called “phosphoinositide-motive force” is not essential (43). Nonetheless, we observed a progressive increase in intracellular PS in C. albicans mutants with decreasing plasma membrane PI(4)P, demonstrating the plasma membrane PI(4)P is critical for PS transport to the plasma membrane. This result indicates that Osh6/7 counter-transporters account for roughly half of the plasma membrane PS and suggests that the remaining PS may be delivered to the plasma membrane via vesicular traffic.

In S. cerevisiae, Stt4 and Ypp1 and Efr3 and Ypp1 colocalize in cortical patches (18), and the latter interaction has also been observed by bimolecular fluorescence complementation (44). In mammalian cells and fission yeast, Stt4, Ypp1, and Efr3 also form a complex observed by coimmunoprecipitation and colocalization (8, 19, 45, 46). Here, we show that all three Stt4 PI-4-kinase complex proteins localize to cortical patches during budding and hyphal growth, yet we did not observe substantial colocalization between Ypp1 and Stt4 proteins, although each protein was critical for the cortical patch localization of the other protein. This suggests that Ypp1 is important for targeting and/or stabilization of the Stt4 PI-4-kinase, as has been observed in S. cerevisiae (18). Interestingly, although Efr3 is important for cortical patch localization of Ypp1 and Stt4 proteins, it is not absolutely required as in S. cerevisiae (18). These results suggest that Efr3 facilitates targeting and/or stabilization of the complex, but that plasma membrane PI-4-kinase activity is not strictly dependent on it.

Cell wall defects in mutants lacking the PS synthase Cho1, which have little to no PS, are in some respects similar to those of *stt4* deletion mutant cells. For example, a C. albicans *cho1* deletion mutant also had a thicker cell wall and exhibited increased sensitivity to the antifungal drug caspofungin (27). However, this *cho1* mutant had a dramatic increase in cell wall chitin levels (27, 30, 47), in contrast to a mutant lacking plasma membrane PI(4)P. Interestingly, both *stt4* and *cho1* deletion mutants exhibited an increase in exposed cell wall β(1,3)-glucan, with the latter mutant having a roughly 10-fold-greater increase in this polysaccharide (30) than the *stt4* mutant. However, given that a *cho1*Δ/*cho1*Δ::*CHO1* strain with >50% reduction in PS levels had no increase in unmasked β(1,3)-glucan (27, 28), it is unlikely that the cell wall defects observed in the mutant lacking plasma membrane PI(4)P is due to the reduced PS levels.

The importance of lipids with respect to C. albicans virulence has been challenging to determine, as a number of lipids are essential for viability and cell growth. One lipid that has been shown to be critical for pathogenicity in a range of different fungi is the sphingolipid glucosylceramide (48–51). Furthermore, mutants lacking either the PS synthase Cho1 or PS decarboxylases (Psd1 and Psd2) were avirulent in murine models of systemic candidiasis and oropharyngeal candidiasis (27, 29). However, both of these mutants grew substantially more slowly than wild-type strains and exhibited an ∼50% reduction in phosphatidylethanolamine (PE) levels (27). Expression of a heterologous serine decarboxylase revealed that the observed virulence defects, as well as β(1,3)-glucan unmasking, were in fact due to the ethanolamine auxotrophy of these mutants (29). Using an *in vitro* assay for host pathogen interactions, an ∼2-fold reduction in macrophage lysis was observed with an *stt4* mutant (17), suggesting that this kinase may be important for virulence. Similarly, the filamentation defect and increased exposure of cell surface β(1,3)-glucan in the plasma membrane PI-4-kinase deletion mutant indicated that PI(4)P has a role in virulence.

Interestingly, a recent study by Dunker et al. showed that rapid proliferation can compensate for the absence of filamentation in a murine model of systemic candidiasis, directly challenging the idea that filamentation is strictly required for virulence (37). Of the three Stt4 PI-4-kinase complex mutants, *stt4*, and to a lesser extent *ypp1*, exhibited virulence defects in a murine model of systemic candidiasis. Given that all Stt4 PI-4-kinase complex mutants are defective in filamentation, this decrease in virulence is unlikely to be attributable to a defect in morphogenesis. Indeed, elongated *stt4* mutant cells were observed after long incubation times with serum or kidney homogenate, and hyphal gene induction was not substantially different from the wild-type strain after 2 h incubation in serum. Furthermore, in a murine model for oropharyngeal candidiasis, all Stt4 PI-4-kinase complex mutants were able to filament. Our results, however, indicate a correlation between PI(4)P at the plasma membrane and systemic candidiasis, given that the *ypp1* mutant and the *stt4* mutant have a 3-fold or greater decrease in plasma membrane PI(4)P. As the Stt4 PI-4-kinase complex mutants all have altered cell wall integrity, substantial alterations in the level of plasma membrane PI(4)P lead to corresponding changes in the cell wall, in particular, exposure of masked β(1,3)-glucan, which is recognized by host immune cells, ultimately contributing to the reduction in virulence in the systemic candidiasis model. Consistent with this explanation, the *stt4* deletion mutant exhibited little to no defect in an OPC infection assay, in which the host immune response is less critical. It will be interesting to further analyze the cell wall defects in these Stt4 PI-4-kinase complex mutants given their different levels of plasma membrane PI(4)P, which will be useful tools in dissecting how plasma PI(4)P regulates the cell wall during fungal infection.

## MATERIALS AND METHODS

### Strain and plasmid construction.

Standard methods were used for C. albicans cell culture and for molecular and genetic manipulations. Strains were grown in yeast extract-peptone dextrose (YEPD) at 30°C unless otherwise indicated, and induction of filamentous growth was carried out with 50% serum at 37°C for 90 and/or 120 min. To determine doubling times, cells were grown in YEPD medium at 30°C and optical density was followed over 8 h of logarithmic growth. For induction with kidney homogenates, kidneys from male C57BL/6 mice were aseptically removed and Dounce homogenized with sterile phosphate-buffered saline (PBS; 0.4 g/mL), and this homogenate was diluted 1:1 with logarithmically growing C. albicans strains in YEPD. Serial dilutions of the different strains on YEPD plates containing Congo red (400 μg/mL), calcofluor white (25 μg/mL), caspofungin (50 and 125 ng/mL), and fluconazole (10 μg/mL) were examined after 2 to 3 days of incubation at 30°C (52). The strains and plasmids used are listed in Table S1A and B, respectively, and the oligonucleotides used are listed in Table S1C. All strains are based on the BWP17 background (53). The *efr3*Δ/*efr3*Δ and *ypp1*Δ/*ypp1*Δ strains were generated by homologous recombination. Each copy was replaced by either *HIS1* or *URA3* using knockout cassettes generated by amplification from pGemHIS1 and pGemURA3 (53) with the primers CaEfr3pKO/CaEfr3mKO and CaYpp1pKO/CaYpp1mKO. In order to generate prototrophic strains, the pExpARG plasmid was linearized with StuI and integrated into the *RPS1* locus. pExpARG-pEFR3-EFR3 and pExpARG-pYPP1-YPP1 plasmids were constructed by amplification of gDNA using primers with a unique XhoI site at the 5′ ends and a unique NotI site at the 3′ ends, with 1 kb upstream and downstream of the respective open reading frames (ORFs). These fragments were subsequently cloned in pExpARG, yielding pExpARG-pEFR3-EFR3 and pExpARG-pYPP1-*YPP1*, respectively. Finally, pExpARG-pEFR3-*EFR3* and pExpARG-*YPP1*-*YPP1* were integrated into the *RPS1* locus, yielding the *efr3*Δ/*efr3*Δ *RPS1*::*EFR3p*-*EFR3* and *ypp1*Δ/*ypp1*Δ *RPS1*::*YPP1p*-*YPP1* recovery strains, respectively.

10.1128/mbio.03873-21.10 TABLE S1 Strains, plasmids, and primers used in this study. Download Table S1, PDF file, 0.2 MB. Copyright © 2022 Garcia-Rodas et al. 2022 Garcia-Rodas et al. https://creativecommons.org/licenses/by/4.0/ This content is distributed under the terms of the Creative Commons Attribution 4.0 International license.

To generate mutants, we first generated an *stt4*-DAmP (decrease abundance by mRNA perturbance) allele by integration of the *SAT1* gene (via amplification of pFASAT1 [54] with the primers CaSATDAmPpS1 and CaSATDAmPmS2) 5′ of the *STT4* stop codon. The remaining *STT4* copy was replaced with *URA3* using a knockout cassette generated from amplification of pGemURA3 (53) with primers CaStt4pKO and CaStt4mKO. As the *stt4*Δ/*stt4*DAmP strain behaved identically to the wild type, we next generated an *stt4*Δ/*stt4*Δ strain (PY4377) by replacing the allele *stt4*-DAmP with *HIS1* using a knockout cassette amplified from pGemHIS1 (53) with the primers CaStt4pKO and CaSATDAmPmKO. The *URA3* gene of the *stt4*Δ/*stt4*Δ strain (PY4377) was then replaced with *SAT1*, which was amplified from pGFP-Nat (55) using the primers CaURApSAT and CaURAmSAT, so that *URA3* could be subsequently integrated at the *RPS1* locus for murine HDC assays. To generate this URA^+^ strain, pExpURA3 (56) was linearized and integrated in the *RPS1* locus, yielding PY5040, which was subsequently rendered ARG^+^ by transformation with linearized pExpARG plasmid, yielding PY5111. In order to reintegrate *STT4* at the *STT4* locus, we generated a pSTT4-STT4-STT4t cassette in which unique AscI and PmeI sites were inserted into the *STT4* terminator (623 bp 3′ of the stop codon) by site-directed mutagenesis using the primers CaStt4term_mAscIPmeI and CaStt4term_pAscIPmeI. Subsequently, *ARG4* was amplified from pFAARG4 using the primers CaARG4AscI-S1 and CaARG4PmeI-S2, yielding plasmid pExpARG-STT4-STT4-STT4t::ARG4. To generate the recovered strain, the pSTT4-STT4-STT4t::ARG4 fragment (digested by XhoI and NotI) from this plasmid was used to replace *stt4*::*HIS1* in PY5040, resulting in *stt4*Δ/*stt4*Δ::STT4, PY5119. To generate a prototroph recovered strain, *HIS1* was added back using linearized pGemHIS1, resulting in PY5131. To generate the hypomorph *stt4* mutant, which encodes Stt4[G1810D], site-directed mutagenesis was carried out with pExpARG-pSTT4-STT4-STT4t::ARG4 using primers CaStt4G1810DpEcoRV and CaStt4G1810DmEcoRV, resulting in pExpARG-STT4p-STT4*-STT4t::ARG4, which was subsequently digested with XhoI and NotI and transformed into PY5040, resulting in PY5757.

Plasmids containing the phospholipid reporters for plasma membrane PI(4)P, Golgi PI(4)P, PI(4,5)P_2_, and PS, pExpARG-pADH1-GFP-(PH^OSH2[H340R]^)_2_-GFP, pExpARG-pADH1-PH^FAPP1[E50A, H54A]^-GFP (13), pExpARG-pADH1-GFP-PH^Plcδ^-PH^Plcδ^-GFP (14), and pExpARG-pACT1-GFP-yeLactC2 (36), respectively, were linearized and integrated into the *RPS1* locus. For expressing these reporters in the Stt4[G1810D] strain (PY5757), the *ARG4* gene was replaced with *SAT1* using the primers CaArgExchS1 and CaArgExchS2 and pFASAT1 (54). To generate fluorescent-protein fusions with Stt4, Efr3, and Ypp1, either 3× mScarlet or mTurquoise2 was amplified using primers CaStt4pXFPS1 and CaStt4mXFPS2, CaEr3pXFPS1 and CaEr3mXFPS2, and CaYpp1pXFPS1 and CaYpp1mXFPS2, respectively, and plasmids pFA-3×-mSc-ARG4 (57), pFA-3×-mSc-CdHIS1, and pFA-mTurq2-ARG4 (C. Puerner, M. Bassilana, and R. A. Arkowitz, unpublished data).

### Southern blot analyses, RT-PCR, qRT-PCR, and chitin staining.

For Southern analysis, EcoRV-digested gDNA was separated on a 1% agarose gel, transferred to a nylon membrane, and fixed by UV cross-linking as described elsewhere (36). The hybridization probes were generated by PCR using primers (for *STT4*, CaStt4p5199 and CaStt4m5543; for URA3, CaUra3pXhoI and CaURA3m81) and an Amersham ECL Direct nucleic acid labeling and detection system kit (GE Healthcare UK Ltd., Little Chalfont, Buckinghamshire, UK) following the manufacturer’s instructions. For RT-PCR and quantitative RT-PCR (qRT-PCR), primers used (Genep-TM and Genem-TM) are listed in Table S1C, and RNA extraction was carried out using a Master Pure yeast RNA extraction purification kit (Epicentre) from budding cells and cells incubated with serum. For RT-PCR actin amplification, 30 cycles were used, and 32 cycles were used to amplify *EFR3*, *YPP1*, *STT4*, **PIK1*α*, *MSS4*, *SAC1*, and the mScarlet gene. qRT-PCR analyses were carried out as previously described (36) with indicated primers (Genep-TM and Genem-TM; Table S1C). For chitin staining exponentially growing cells were fixed, stained with 25 μg/mL calcofluor white solution, and imaged using a spinning disk confocal microscope (58).

### Quantitation of cell wall components using flow cytometry.

Logarithmically growing strains were stained for exposed β(1,3)-glucan using an anti-β(1,3)-glucan monoclonal antibody (MAb) (400-2; Biosupplies, Australia) as the primary antibody and a donkey anti-mouse IgG (heavy plus light chain [H+L]) as the secondary antibody, conjugated to Alexa Fluor 568 (A10037; Thermo Fisher, France), essentially as described elsewhere (28). Antibody dilutions of 1:600 (primary) and 1:500 (secondary) were used. For total chitin, mannan, and glucan, calcofluor white (fluorescent brightener 28 M2R; Sigma), concanavalin A (ConA)-tetramethylrhodamine (11540176; Thermo Fisher, France), and aniline blue soluble sodium salt (10656822; Thermo Fisher, France), were used at concentrations of 25 μg/mL, 50 μg/mL, and 50 μg/mL, respectively. Cells were fixed with 4% paraformaldehyde (PFA) in PBS for all analyses and washed prior to staining. Incubation of cells with ConA was for 30 min, and cells were subsequently washed. Flow cytometry was carried out on a BD LSRFortessa Sorp cell analyzer using 355-nm and 561-nm laser lines with Hoechst blue (450/50 nm) and phycoerythrin (PE)-Texas Red (600 nm long pass mirror; 610/20 nm) filters, respectively. Data were obtained from 100,000 gated events per strain, from 3 independent experiments.

### Microscopic analyses.

For colony morphology analysis plates were incubated for 3 to 6 days prior to imaging (36). mScarlet and mTurquoise fusions were imaged as described elsewhere (57, 58) with 17 0.5-μm z-sections. Quantitation of plasma membrane and internal mean signals was performed on central z-sections using the Matlab program Hyphal-Polarity (13). Ratios of plasma membrane to internal signals were normalized to the mean wild-type ratio. Plasma membrane fractions of PI(4)P, PI(4,5)P_2_, and PS were calculated as the ratio of plasma membrane signal to total signal, which was then normalized to the mean wild-type ratio. To represent these values between 1 and 0, 0.5 was subtracted from the normalized ratio (the Matlab program detects first signal going in from the region of interest [ROI] above background, which is the cytoplasm when there is no plasma membrane localization; hence, a value of 0.5 is the absence of plasma signal) and then multiplied by 2 so that the value for the wild-type plasma membrane fraction is 1 and the value for no plasma membrane signal is 0. Huygens professional software version 18.04 (Scientific-Volume Imaging) was used for deconvolution of image z-stacks using the appropriate settings for the microscope and excitation source. The signal-to-noise ratio was set to 10, and the background detection was set to auto, unless otherwise stated.

### Virulence assays.

HDC was induced in 10 BALB/c mice (Charles River, Italy) per group by injecting the lateral tail vein with an inoculum of 5 × 10^5^ cells (59). Animal body weight was monitored daily, and animals were sacrificed by cervical dislocation when they had lost more than 20% of their weight. OPC was induced in mice that had been immunosuppressed with cortisone acetate using 7 to 8 mice per strain as previously described (60). A Vectra Polaris slide scanner was used to scan histopathology of murine tongue thin sections, stained with periodic acid-Schiff stain.

### Ethics statements.

All OPC animal experiments were approved by the Institutional Animal Care and Use Committee (IACUC) of the Lundquist Institute at Harbor-UCLA Medical Center. All HDC animal procedures were approved by the Bioethical Committee and Animal Welfare of the Instituto de Salud Carlos III (CBA2014_PA51) and of the Comunidad de Madrid (PROEX 330/14) and followed the current Spanish legislation (Real Decreto 53/2013) along with Directive 2010/63/EU.

### Statistical analysis.

Differences in mean signals, ratios, and percentages of filaments were analyzed by *t* test, and survival experiments with mice were analyzed by the Kaplan-Meier method (log-rank test) with GraphPad Prism 8 (GraphPad, La Jolla, CA).

### Transmission electron microscopy.

Budding cell samples were processed for electron microscopy and images were acquired as described elsewhere (61). Cell wall thickness was measured from electron micrographs at 3 or 4 locations around the mother cell and averaged.
