# Supplementary figures and images for: Plasma Membrane Phosphatidylinositol-4-Phosphate Is Not Necessary for Candida albicans Viability yet Is Key for Cell Wall Integrity and Systemic Infection
Source: mBio. 2022 Feb 15;13(1):e03873-21. doi: 10.1128/mbio.03873-21 (PMC8942462; doi:10.1128/mbio.03873-21)

A

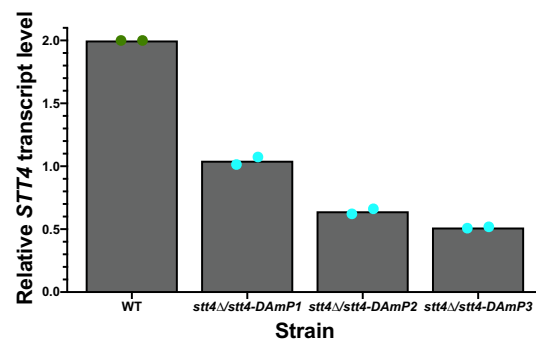

B

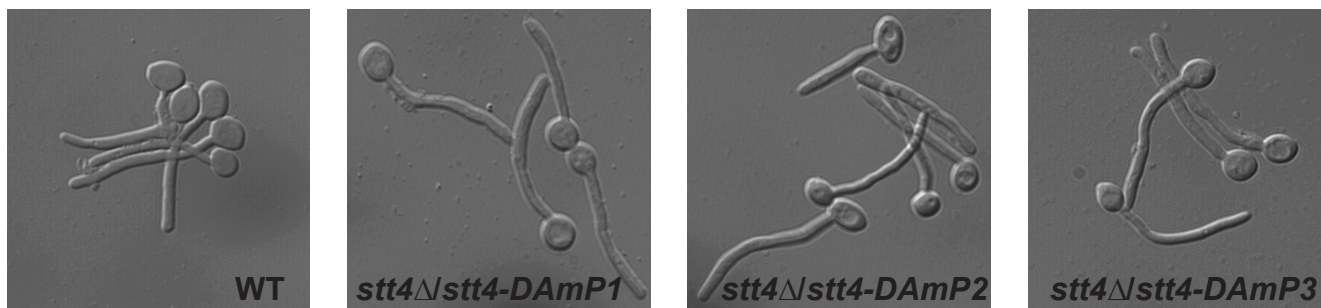

C

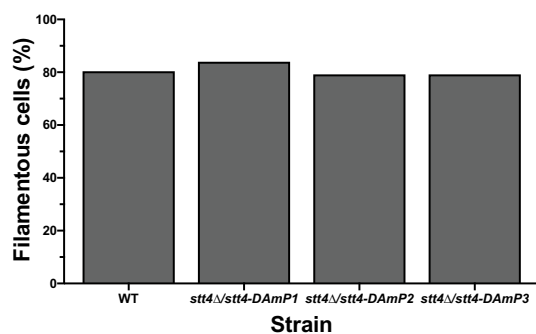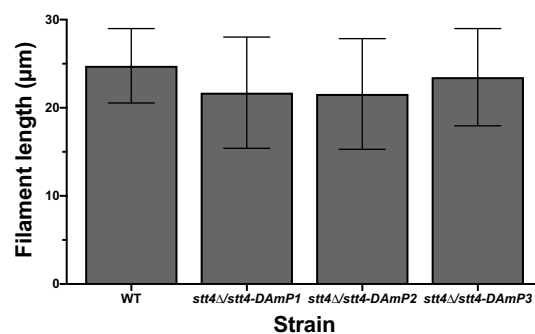

D

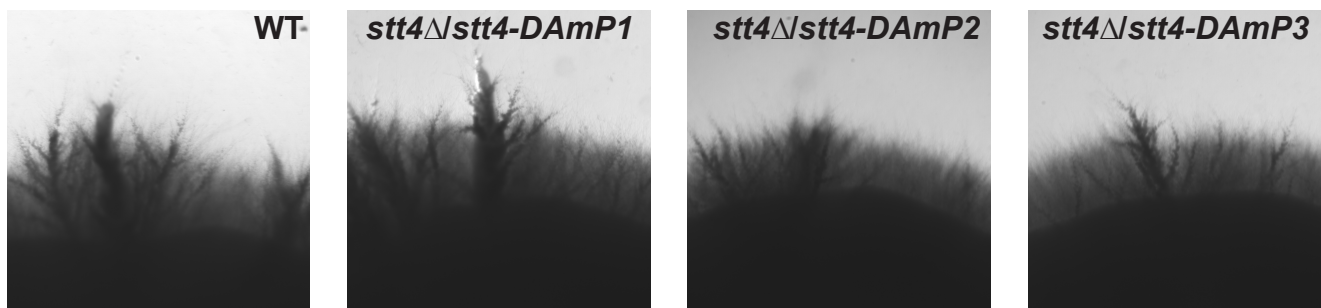

Supplement: FIG S1 [file mbio.03873-21-sf001.pdf]

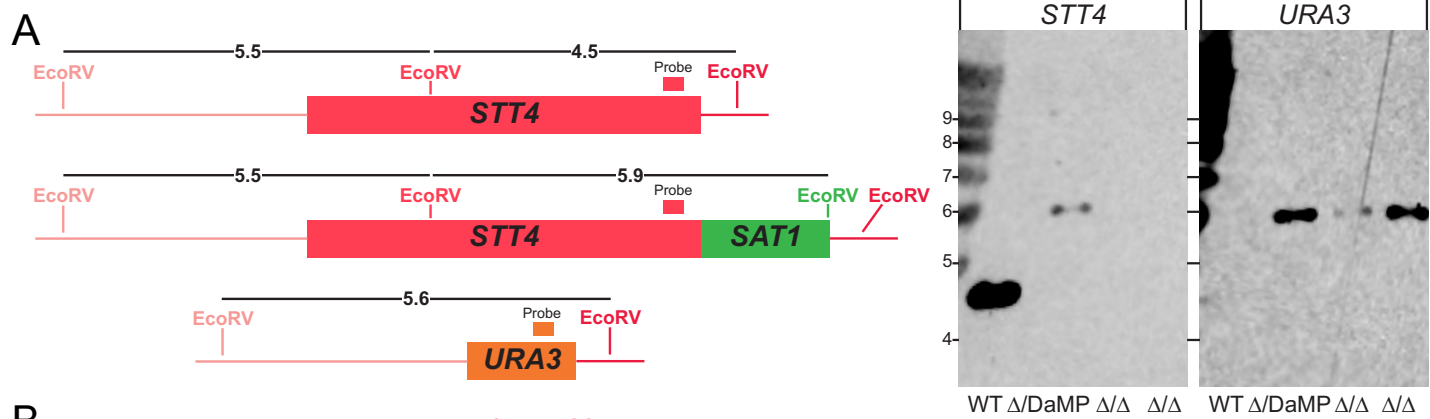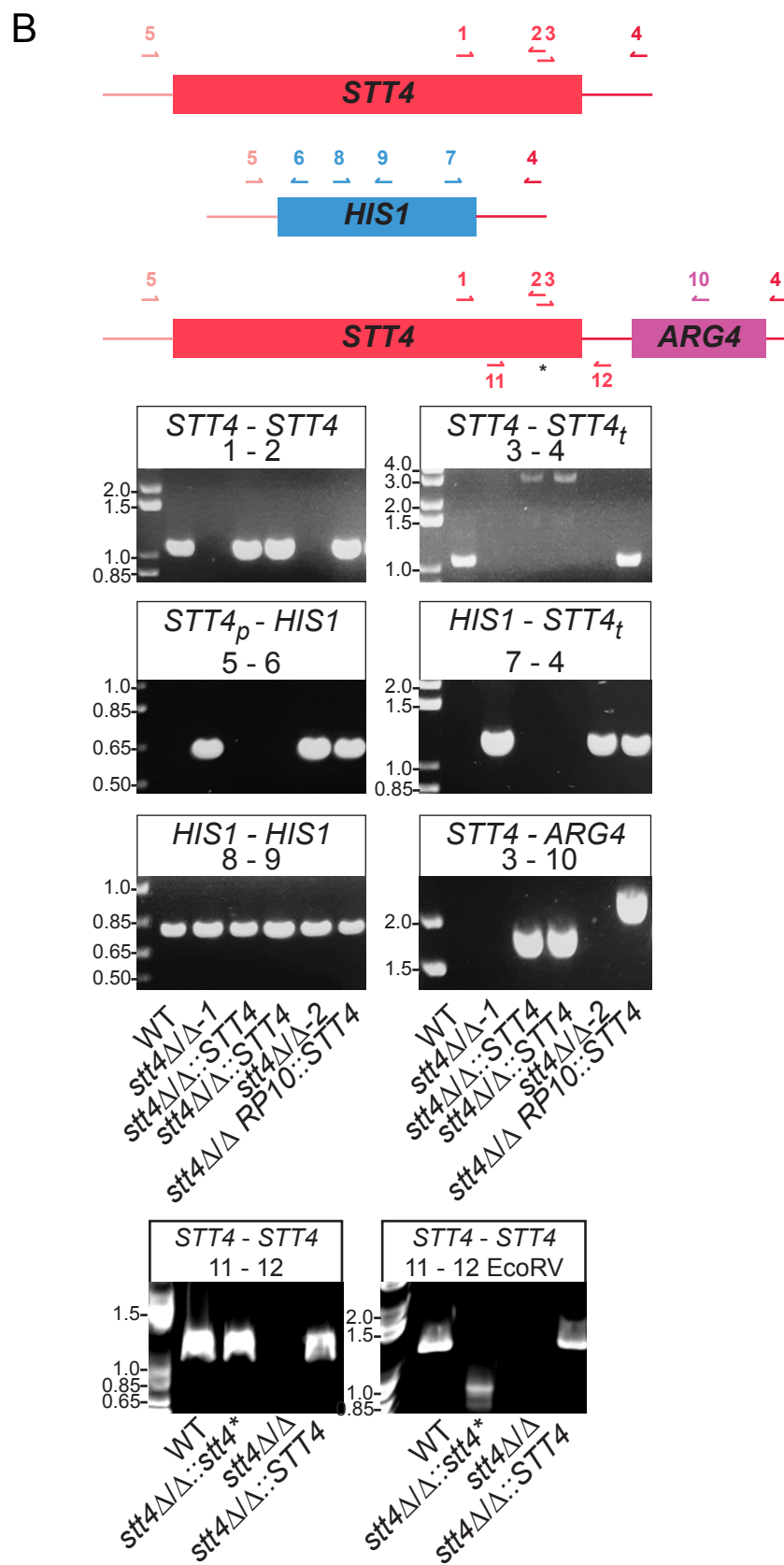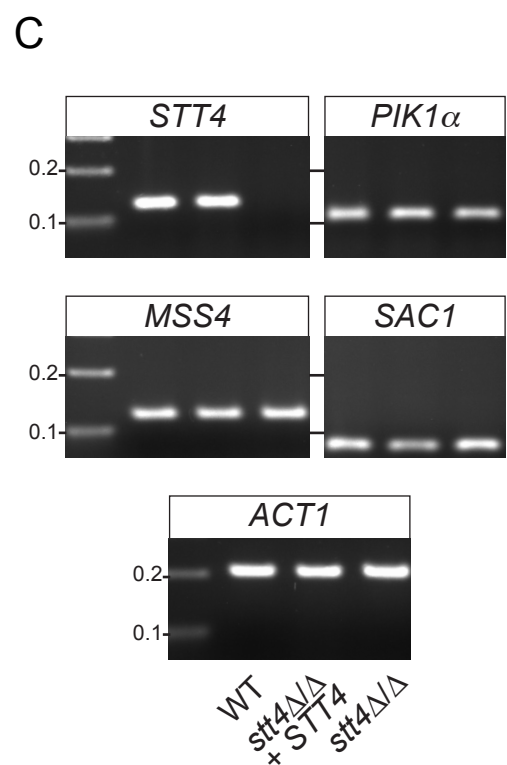

Supplement: FIG S2 [file mbio.03873-21-sf002.pdf]

A

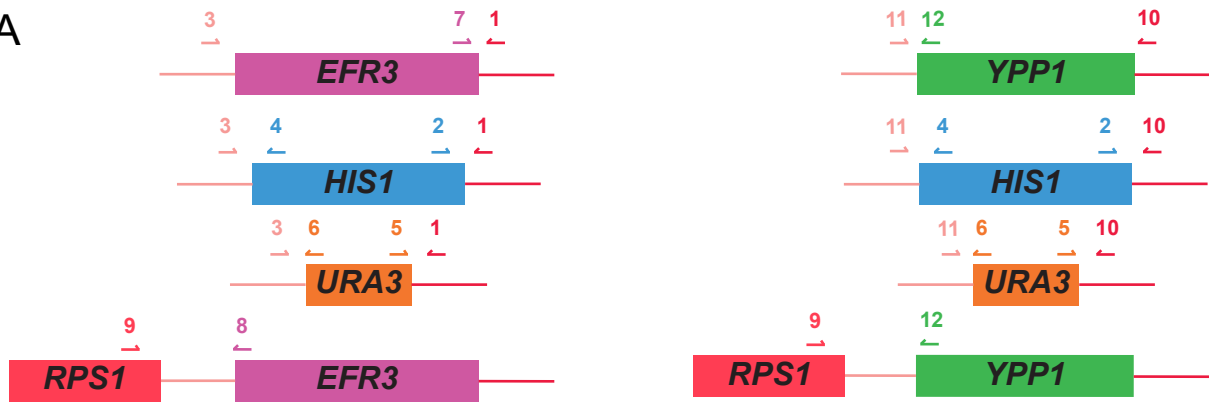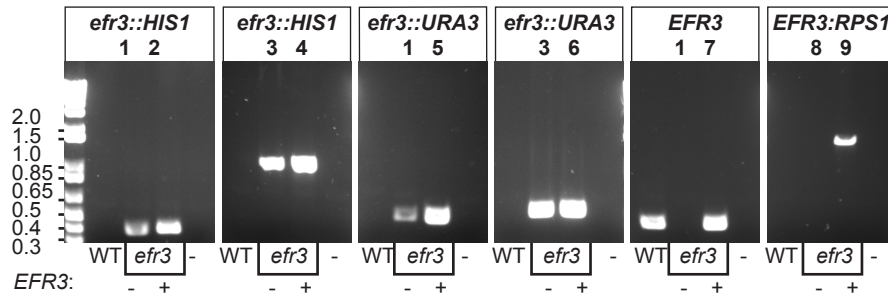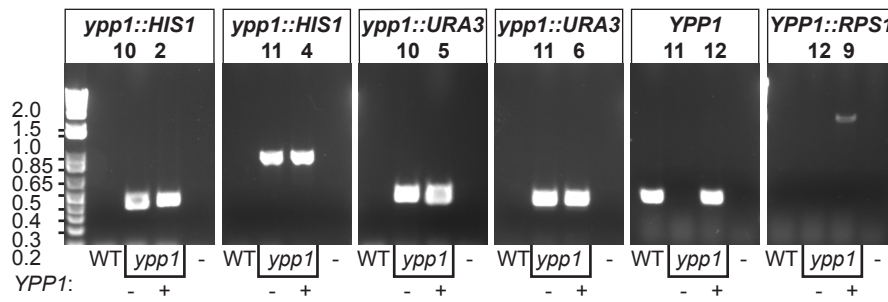

B

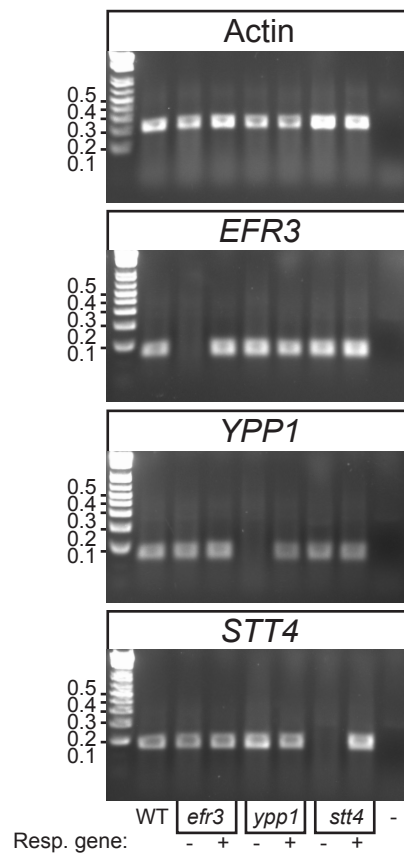

Supplement: FIG S3 [file mbio.03873-21-sf003.pdf]

A

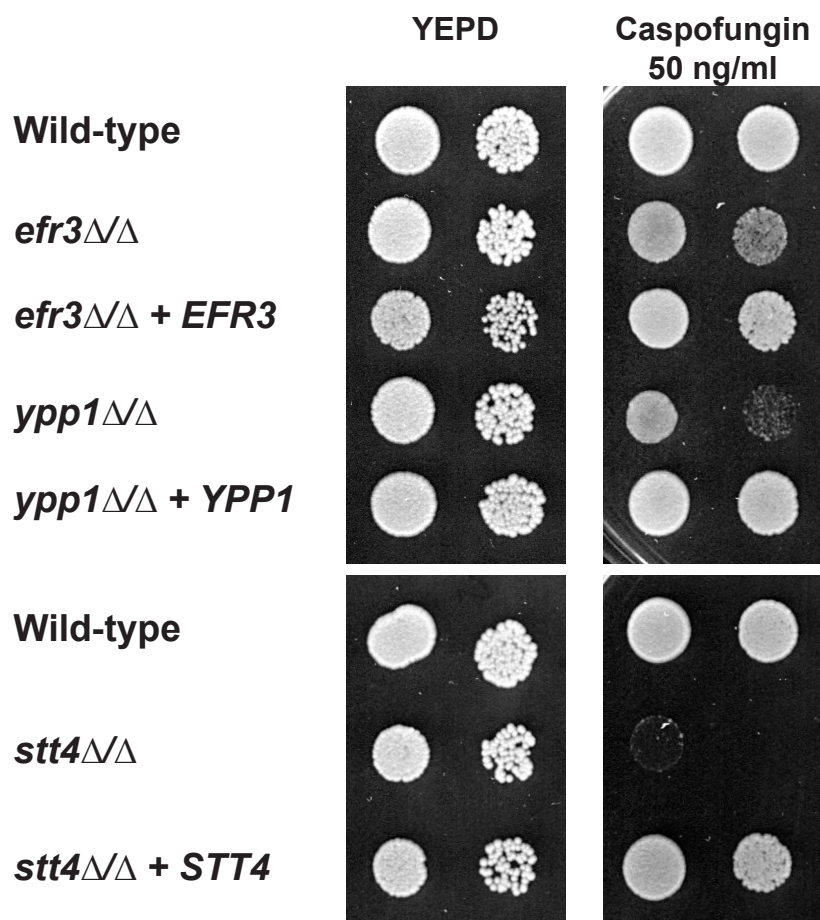

B

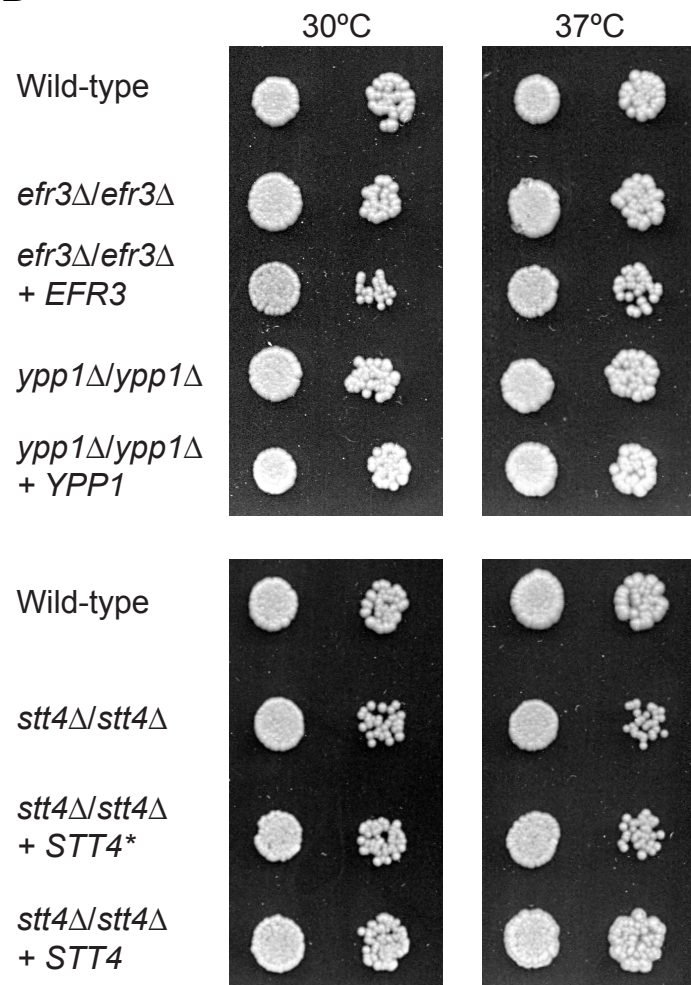

Supplement: FIG S4 [file mbio.03873-21-sf004.pdf]

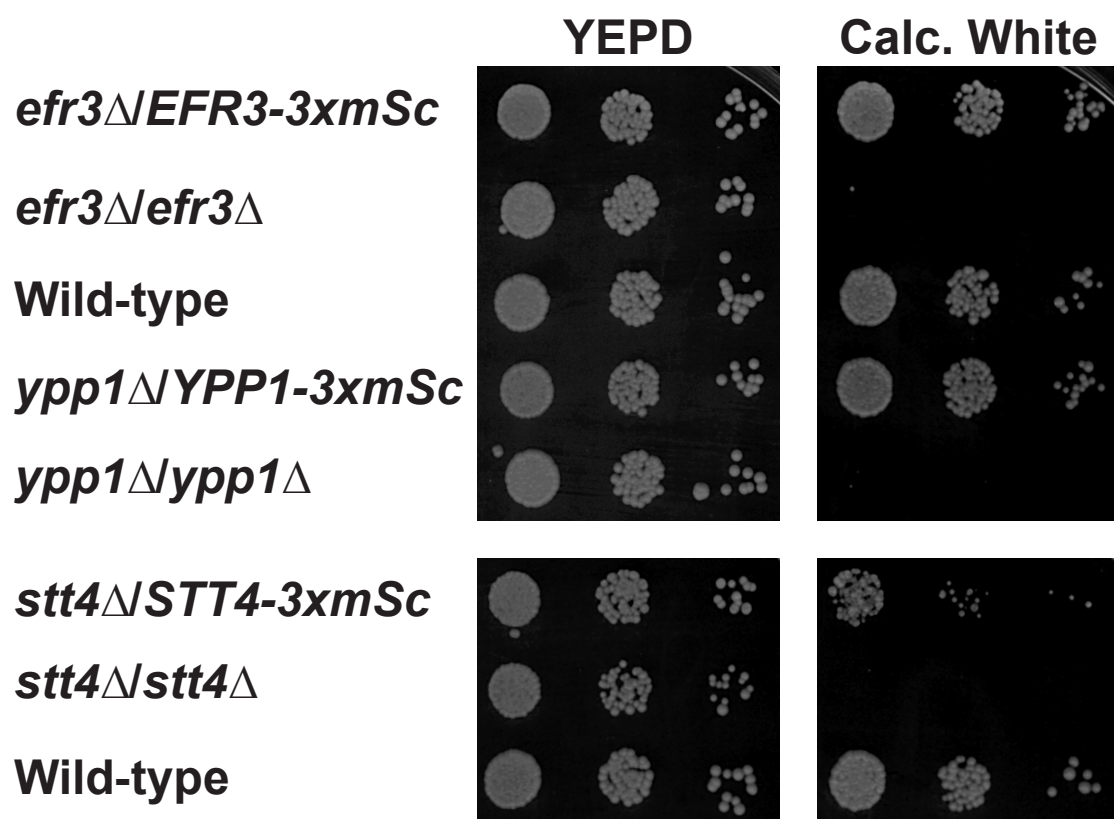

Supplement: FIG S5 [file mbio.03873-21-sf005.pdf]

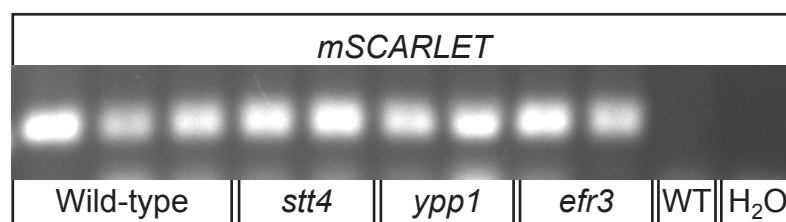

YPP1-3xmSc: + + +

EFR3-3xmSc: + + +

STT4-3xmSc: + + +

Supplement: FIG S6 [file mbio.03873-21-sf006.pdf]

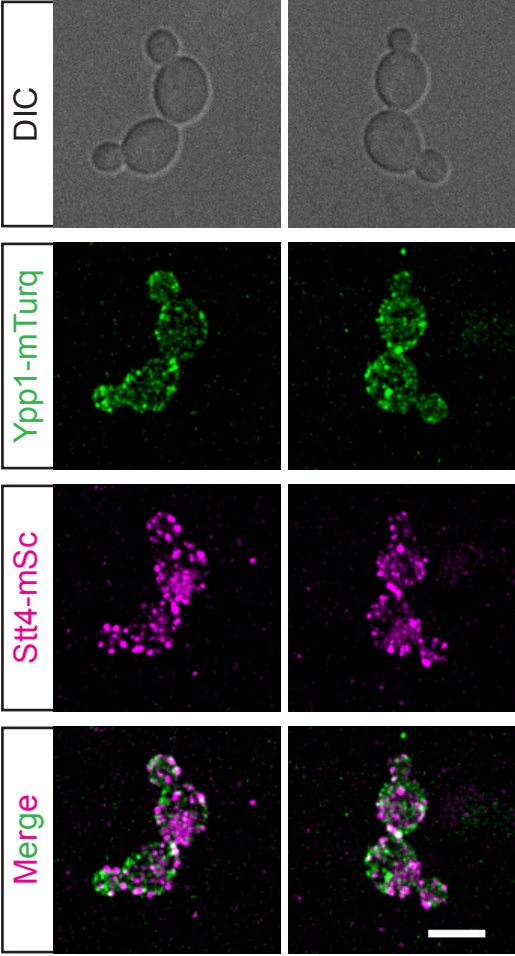

Supplement: FIG S7 [file mbio.03873-21-sf007.pdf]

## FCS

WT

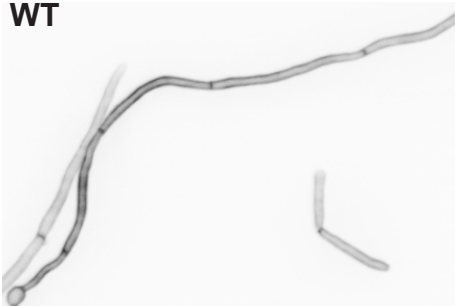

*stt4*

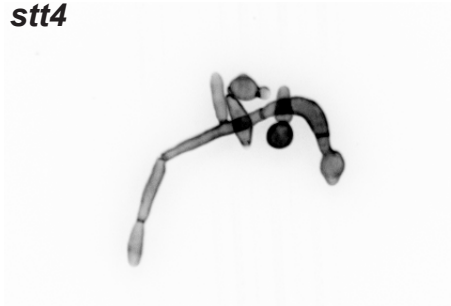

*stt4* + *STT4*

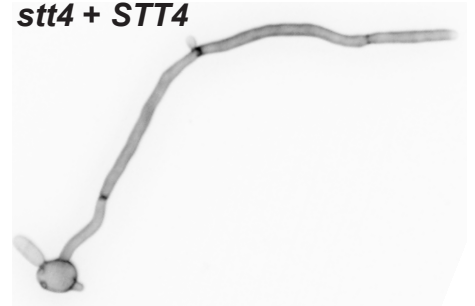

## Kidney homogenate

WT

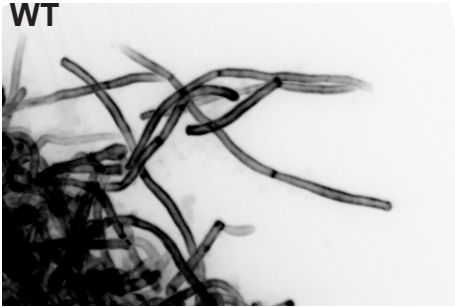

*stt4*

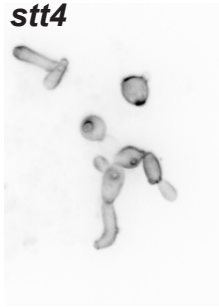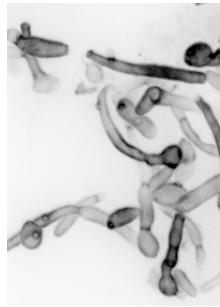

*stt4* + *STT4*

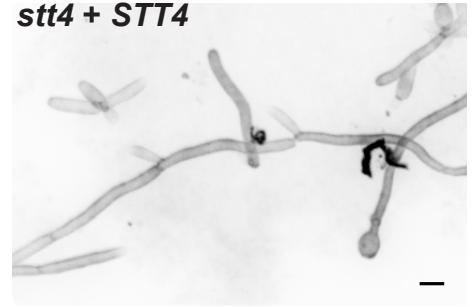

Supplement: FIG S8 [file mbio.03873-21-sf008.pdf]

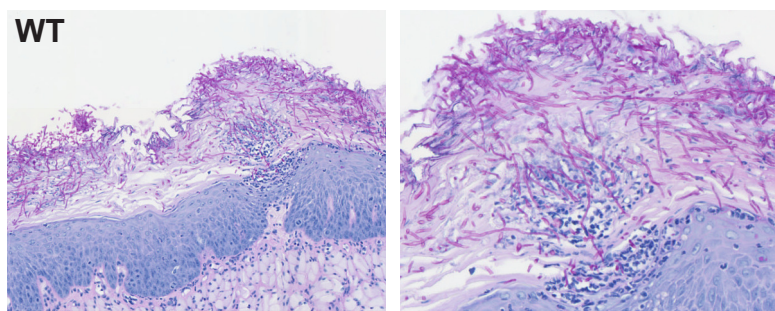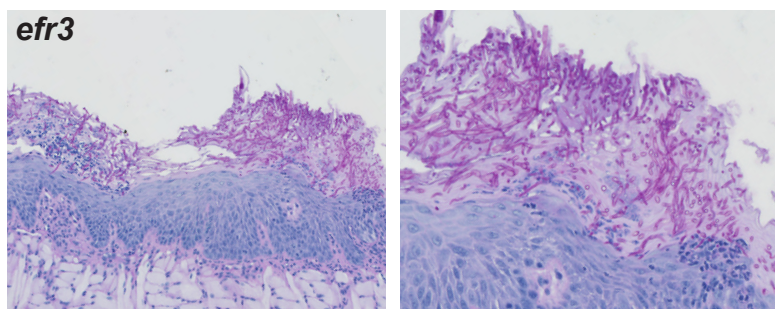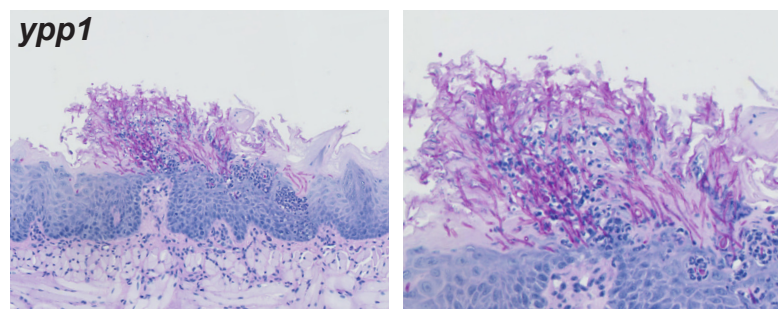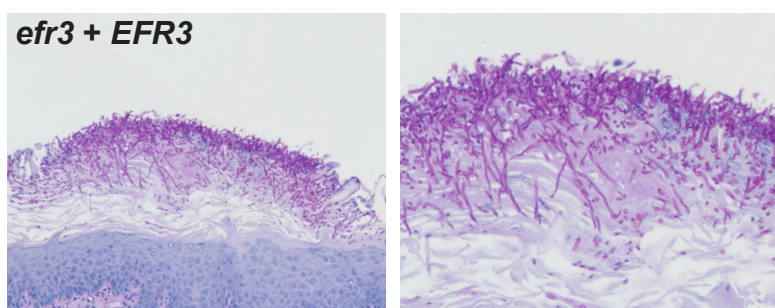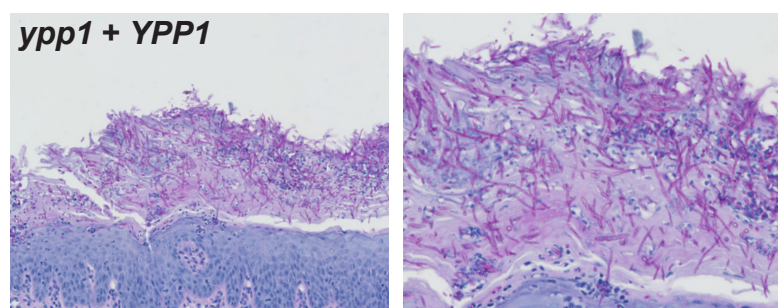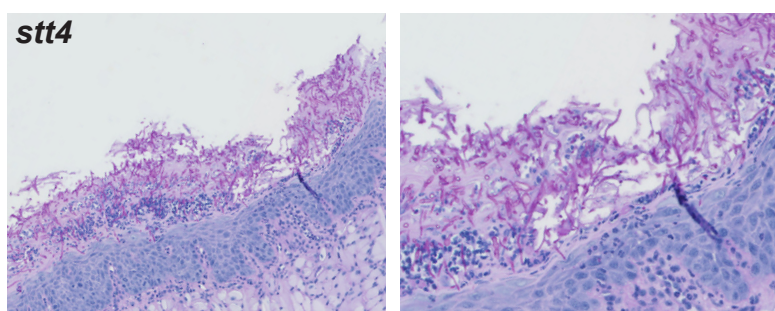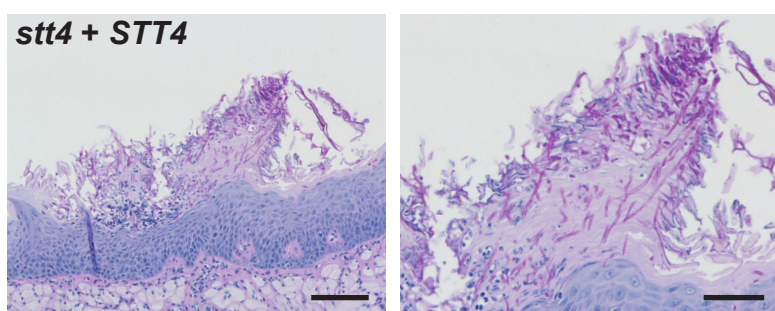

Supplement: FIG S9 [file mbio.03873-21-sf009.pdf]
