# Supplementary material for: Plasma Membrane Phosphatidylinositol-4-Phosphate Is Not Necessary for Candida albicans Viability yet Is Key for Cell Wall Integrity and Systemic Infection
Source: mBio. 2022 Feb 15;13(1):e03873-21. doi: 10.1128/mbio.03873-21 (PMC8942462; doi:10.1128/mbio.03873-21)
Supplement: TABLE S1 [file mbio.03873-21-st001.pdf]

Table S1. Strains, plasmids and primers used in this study.
A) Strains

Strain	GENOTYPE	SOURCE
BWP17	*ura3*Δ::λimm434/*ura3*Δ::λimm434 *his1*Δ::*hisG*/*his1*Δ::*hisG* *arg4*Δ::*hisG*/*arg4*Δ::*hisG*	(1)
PY173	*ade2*Δ::*hisG*/*ade2*Δ::*hisG* *ura3*Δ::λimm434/*ura3*Δ::λimm434 *his1*Δ::*hisG*/*his1*Δ::*hisG* *arg4*Δ::*hisG*/*arg4*Δ::*hisG* *ENO1*/*eno1*::*ENO1*-tetRScHAP4AD-3xHA-ADE2	(2)
PY1206	Same as BWP17 with *RPS1*::*ARG4*-p*ADH1*-GFP-(PH$^{Plc\partial}$)$_2$-GFP	(2)
PY2578	Same as PY173 with *RPS1*::*ARG4*-p*ADH1*-PH$^{FAPP1[E50A,H54A]}$-GFP	(3)
PY2626	Same as PY173 with *RPS1*::*ARG4-pACT1*-GFP-(PH$^{OSH2[H340R]}$)$_2$-GFP	(3)
PY3239	Same as BWP17 with *RPS1*::*ARG4*-p*ACT1*-GFP-yeLactC2	(4)
PY3857	Same as BWP17 with *efr3Δ::HIS1/EFR3*	This study
PY3859	Same as BWP17 with *ypp1Δ::HIS1/YPP1*	This study
PY3915	Same as BWP17 with *efr3Δ::HIS1/efr3Δ::URA3*	This study
PY3918	Same as BWP17 with *ypp1Δ::HIS1/ypp1Δ::URA3*	This study
PY3933	Same as PY3915 with *RPS1*::*ARG4*-p*ADH1*-PH$^{FAPP1[E50A,H54A]}$-GFP	This study
PY3935	Same as PY3915 with *RPS1*::*ARG4*-p*ADH1*-GFP-(PH$^{Plc\partial}$)$_2$-GFP	This study
PY3947	Same as PY3915 with *RPS1*::*ARG4*-p*ACT1*-GFP-(PH$^{OSH2[H340R]}$)$_2$-GFP	This study
PY3950	Same as PY3918 with *RPS1*::*ARG4*-p*ACT1*-GFP-(PH$^{OSH2[H340R]}$)$_2$-GFP	This study
PY3951	Same as PY3918 with *RPS1*::*ARG4*-p*ADH1*-PH$^{FAPP1[E50A,H54A]}$-GFP	This study
PY3958	Same as PY3918 with *RPS1*::*ARG4*-p*ADH1*-GFP-(PH$^{Plc\partial}$)$_2$-GFP	This study
PY4033	Same as PY3918 with *RPS1*::*ARG4*	This study
PY4036	Same as PY3915 with *RPS1*::*ARG4*	This study
PY4039	Same as PY3915 with *RPS1*::*ARG4-EFR3pEFR3*	This study
PY4040	Same as PY3918 with *RPS1*::*ARG4-YPP1pYPP1*	This study
PY4124	Same as PY3915 with *RPS1*::*ARG4-pACT1*-GFP-yeLactC2	This study
PY4131	Same as PY3918 with *RPS1*::*ARG4-pACT1*-GFP-yeLactC2	This study
PY4330	Same as BWP17 with *stt4Δ::URA3/STT4*	This study
PY4335	Same as BWP17 with *STT4*::*SAT1* (*stt4-DAmP*)/*STT4*	This study
PY4339	Same as PY4335 with *stt4Δ::URA3/STT4*::*SAT1* (*stt4-DAmP1*)	This study
PY4340	Same as PY4335 with *stt4Δ::URA3/STT4*::*SAT1* (*stt4-DAmP2*)	This study
PY4341	Same as PY4335 with *stt4Δ::URA3/STT4*::*SAT1* (*stt4-DAmP3*)	This study
PY4377	Same as PY4339 with *stt4Δ::HIS1/stt4Δ::URA3*	This study
PY4378	Same as PY4339 with *stt4Δ::HIS1/stt4Δ::URA3*	This study
PY4414	Same as PY4377 with *RPS1::ARG4*	This study
PY4433	Same as PY4377 with *RPS1::ARG4-STT4pSTT4*	This study
PY4861	*ura3Δ::λ imm434/ura3Δ::λ imm434 his1::hisG/HIS1::his1::hisG arg4::hisG/ARG4::URA3::arg4::hisG*	(5)
PY5027	Same as PY4377 with *stt4Δ::HIS1/stt4Δ::SAT*	This study

PY5040	Same as PY5027 with *RPS1*::*URA3*	This study
PY5111	Same as PY5040 with *RPS1*::*ARG4*	This study
PY5119	Same as PY5040 with *stt4::STT4pSTT4-ARG4/stt4Δ::SAT*	This study
PY5131	Same as PY5119 with *his1*::*HIS1*	This study
PY5132	Same as PY5119 with *his1*::*HIS1*	This study
PY5169	Same as PY5040 with *RPS1*::*ARG4-pACT1*-GFP-(PH$^{OSH2[H340R]}$)$_2$-GFP	This study
PY5174	Same as PY5040 with *RPS1*::*ARG4-pACT1*-GFP-yeLactC2	This study
PY5552	Same as PY5040 with *RPS1*::*ARG4-pADH1*-PH$^{FAPP1[E50A,H54A]-}$GFP	This study
PY5554	Same as PY5040 with *RPS1*::*ARG4-pADH1*-GFP-(PH^{Plcδ})$_2$-GFP	This study
PY5574	Same as PY3859 with *YPP1::3x-mSc- ARG4/ypp1Δ::HIS1*	This study
PY5587	Same as BWP17 with *STT4::3x-mSc-cdHIS1/STT4*	This study
PY5599	Same as PY3857 with *EFR3::3x-mSc- ARG4/efr3Δ::HIS1*	This study
PY5601	Same as PY4330 with *STT4::3x-mSc-cdHIS1/stt4Δ::URA3*	This study
PY5757	Same as PY5040 with *stt4::STT4pSTT4*[G1180D]*-ARG4/stt4Δ::SAT*	This study
PY5808	Same as PY5757 with *stt4::STT4pSTT4*[G1180D]*-SAT1/stt4Δ::HIS1*	This study
PY5839	Same as PY5808 with *RPS1*::*ARG4-pACT1*-GFP-(PH$^{OSH2[H340R]}$)$_2$-GFP	This study
PY5903	Same as PY5808 with *RPS1*::*ARG4-pACT1*-GFP-yeLactC2	This study
PY6134	Same as PY5040 with *YPP1::3x-mSc-ARG4*	This study
PY6136	Same as PY3915 with *YPP1::3x-mSc-ARG4*	This study
PY6138	Same as PY3919 with *EFR3::3x-mSc-ARG4*	This study
PY6140	Same as PY5040 with *EFR3::3x-mSc-ARG4*	This study
PY6142	Same as PY3915 with *STT4::3x-mSc-ARG4*	This study
PY6144	Same as PY3919 with *STT4::3x-mSc-ARG4*	This study
PY6193	Same as BWP17 with *STT4::3x-mSc-ARG4*	This study
PY6195	Same as BWP17 with *YPP1::3x-mSc-ARG4*	This study
PY6197	Same as BWP17 with *EFR3::3x-mSc-ARG4*	This study
PY6201	Same as PY5587 with *YPP1::mTurq-ARG4*	This study

B) Plasmids

Plasmid	Source
pExpARG-pADH1-GFP-(PH$^{OSH2[H340R]}$)$_2$-GFP	(3)
pExpARG-pADH1-PH$^{FAPP1[E50A, H54A]}$-GFP	(3)
pExpARG	(3)
pExpARG-pACT1-GFP-yeLactC2	(4)
pExpARG-pADH1-GFP-PH$^{Plc\delta}$-PH$^{Plc\delta}$-GFP	(2)
pGEM-*HIS1*	(1)
pGEM-*URA3*	(1)
pFA-*SAT1*	(6)
pFA-*HIS1*	(6)
pFA-*URA3*	(6)
pFA-*ARG4*	(6)
pFA-*3x-mSc-ARG4*	(7)
pFA-*3x-mSc-cdHIS1*	(C. Puerner, M. Bassilana, and R. A. Arkowitz, in preparation)
pExp*ARG4*-p*EFR3-EFR3*	This study
pExp*ARG4*-p*YPP1-YPP1*	This study
pExp*ARG4*-p*STT4-STT4*	(2)
pExpARG4-pSTT4-*STT4-STT4t::ARG4*	This study
pExpARG4-p*STT4-STT4*-STT4t*::*ARG4*	This study
pFA-*GFPY-NAT*	(8)
pFA-*mTurq-ARG4*	(C. Puerner, M. Bassilana, and R. A. Arkowitz, in preparation)

C) Primers

Primer	Sequence 5'→3'
CaEfr3mKO	cctttttacctagaaaggtattttgacttgggtatctttgcaactgtctctctctcgctttagcttaaacaacaaacgcagcTTTCCCAGTCACGACGTT
CaEfr3pKO	cttttctattggttaattaaatcctgttttgattcaatagtttcttcaacttccagatccaagaatgaatttgtttcaacatTGTGGAATTGTGAGCGGATA
CaEfr3mXFPS2	ggataaaaaaaatgcagtaaatgttcctttttacctagaaaggtattttgacttgggtatctttgcaactgtctctctctcgctttagcTCTGATATCATCGATGAATTCGAG
CaEfr3pXFPS1	gtgttgaataaaaatatgcaaactacagatgttgattcaattttaagtggtcttgaaagtgaagacgaagctgcgtttgttgttGGTGCTGGCGCAGGTGCTTC
CaEfr3m127	CTCTACGAGTGGATGCATA
CaEfr3m3118	CCTCAAACTTACACACTCTTCC
CaEfr3m3183NotI	TATAGCGGCCGCctgcaaataggggactcttaac
CaEfr3p595	GCAAACAACCCACAAAGCA
CaEfr3p1304	CGTCCACTGGAGAACAAAT
CaEfr3p1980	GGATGAGCAATATAGTCAT
CaEfr3p2593	CGAACTGTCGATAGTGTT
CaEfr3p2692	ATGTCAACACATAGGGGTA
CaEfr3pup100	TTCATCAAGAATATCCTT
CaEfr3pup960XhoI	GCCTCGAGgccccactcccactcccccaac
CaStt4mKO	ctatagataaacatacactattaacgcctccactagtaaggaataccattggtaagtctttggaattcatTTTCCCAGTCACGACGTT
CaStt4pKO	cagaaagaacagaaaagctatggattattctgggattacccgtggctcaattcgtgctgaagcccttaagTGTGGAATTGTGAGCGGATA
CaStt4mXFPS2	GGTTGCAGCCAAAAACACGAGCGGATAACAAAGTCCAATGTCAATAATTACTATAGATAAACATACACTATTAACGCCTCCATCTGATATCATCGATGAATTCGAG
CaStt4pXFPS1	CTTTAGAAAATTGATCAAGAAATCGATGGAAAGTTTCTACACTAAAGGTTATGATGAATTCCAAAGACTTACCAATGGTATTCCTTACGGTGCTGGCGCAGGTGCTTC
CaStt4G1810DmEcoRV	GGTGCAGCCTCAAACCTAACCCCATCAGGAACGATATCAAAACAGAACCCAAAATCAATATG
CaStt4G1810DpEcoRV	CATATTGATTTTGGGTTCTGTTTTGATATCGTTCCTGATGGGGTTAGGTTTGAGGCTGCACC
CaStt4m160	GTAATTTGGGAGTGGCCG
CaStt4m4255	CTGATAGCAGGTAATGGGTC
CaStt4m5056	CATGGAAATCAATTGTAAGGC
CaStt4m5130	GGAGCAGTTGCAGTAACT
CaStt4m5543	CCTTTCACACACAACTCTTC
CaStt4m5857	ttgtaacaaaggttgcagcc
CaStt4m6102	CCTTGTGGTTGCTATTTGG
CaStt4m6286	GCTGGTCTTGGGGGGTTGG
CaStt4m6784	ATTTCATATCCAGCAATTATGCC
CaStt4m6924	CCACTTCATTAAACAAGCC

CaStt4m4115KI	CTAGTATCTGAAGGATTCATTGAATGTAACCACACAGTAAATTTACAAATCTCATCATCCATAAAATATAAAAGAATTTTcatagcttttctgttctttctg
CaStt4m4864KI	GATTTTAAATGTCGCCATAAATGGTGCCTTGGCATGTGATTGTAATGGTCTACCCAGTTTACGATTAATATCAATAACAACcatagcttttctgttctttctg
CaStt4m6600NotI	TCTGCGGCCGCcgtggatcacaacattgtg
CaStt4mupSacII	TTAccgcggCATAGCTTTTCTGTTCTTTCTGTTTTCTTG
CaStt4p4100	CATTGCGTACCGATGAAG
CaStt4p4884	GCCATCCAATCCTGATGGGGTTG
CaStt4p5199	GGGAAGAGAAGCTGTTAATGG
CaStt4p5439	GTTCCTGGTGGGGTTAGG
CaStt4p5488	GGAGGTAATGATCAAACTC
CaStt4p5519	GGTTTGAAGAGTTGTGTGTG
CaStt4p5740	GGTTATGATGAATTCCAAAG
CaStt4p5857	ttgtaacaaaggttgcagcc
CaStt4p5974	CGGTCAATAATCTTCGGGATTAAAG
CaStt4pup168	CCCACCACCAACGCAATTATC
CaStt4pup325	caatcacgtgcattctccac
CaStt4pup617S1	GAGACACTTTGATTGAAACTTATTCTTAAATTGGGTTTCTCAAAATATAGGAATTAAAAAGGGGTTTATGTATAGAGTGTTAGgaagcttcgtacgctgcaggtc
CaStt4pupSpeI	GGCactagtCCCGCTCCCAACTAATCAAC
CaStt4term_mAscIPmeI	agaatagaggaagaaaaaaaaagggTTTaaaccatGgcGCGCCagatctcaacgatacttgtacg
CaStt4term_pAscIPmeI	cgtacaagtatcgttgagatctGGCGCgcCatggtttAAAccctttttttttcttcctctattct
CaYpp1mKO	attgatttttatatattctcctttttgatagtctgtttcgctctttgtctattaaagttcatcaaatataaattcccTTTCCCAGTCACGACGTT
CaYpp1pKO	cacatcctattaagttgatcttttattttggcttttaattattttaattcccaacattatagatgtatattgagagttcgTGTGGAATTGTGAGCGGATA
CaYpp1mXFPS2	gctttctgtacatattttattgatttttatatattctcctttttgatagtctgtttcgctctttgtctaTCTGATATCATCGATGAATTCGAG
CaYpp1pXFPS1	gataaaatattattgacgaaaagtttatggagatgtattgaattagaagataaacgaccagttagggaatttatatttgatgaacttGGTGCTGGCGCAGGTGCTTC
CaYpp1m87	TTGTTGGAATTCCAATAC
CaYpp1m3313	ACTTACAAGGCTCATATC
CaYpp1m3577NotI	TATAGCGGCCGCcaaacgaggaacaatcaaagtgtcg
CaYpp1p503	GTGCGGGGAACAATGGAGT
CaYpp1p1173	CAACCCATTGAAAAGTTTC
CaYpp1p1929	GAAGCATCGTCAACGCAAT
CaYpp1p2637	TCCACGTCATCAACATGAT
CaYpp1p3073	GGATATAATAATCCAGAA
CaYpp1pup167	CAGTTTAGCCGGTTGTCC
CaYpp1pup962XhoI	GCCTCGAGgtcacattaactcctatacttgc

CaARG4ExchS1	atgtcacaacaacaagataaacaacctagtgaaaataaatt atggggtggtcgtttcaccggtgccactgatccattgatgg GAAGCTTCGTACGCTGCAGGTC
CaARG4ExchS2	ttaacttaagattgattttaaattttctaattgttttaaaa cggcacttttagcagtaccaccaagagcatctcttctttcc
CaARG4AscI-S1	tctggcgcgccAAGCTTCGTACGCTGCAGGTC
CaARG4PmeI-S2	agggtttaaacTCTGATATCATCGATGAATTCGAG
CaARG4m537	GTGCCCATCTAATAGGTTGAG
CaHIS1mup152	CGAAATGGCCTCCCCTAC
CaHIS1m836	tcgtccattacattaccg
CaHIS1pStop1008	CTTTAGATGGATCGACATTGG
CaHis1p214	gacttgggtataactggg
CaURA3mup270	ggcactacagcaactttc
CaURA3m81	CATAAATTGGTTTTCTTCAGTTCC
CaURA3mSAT1	tataattggccagtttttttcaaataagcattccaaccagc atctctatacctttaccttcaatatctggatcCGTTAGTA TCGAATCGACAGC
CaURA3p751	GAAGGTAAAAGGTATAGAAATGC
CaURA3pSAT1	atgacagtcaacactaagacctatagtgagagagcagaaac tcatgcctcaccagtagcacaacgattatttcgattaGATA TCAAGCTTGCCTCGTC
CaURA3pXhoI	gttgctgtagtgccattgaCtcgAGacactaattctgtatt agtcattcc
CaRPS1p	GAGGATTGAGCGTAAGTAG
CaSATDAmPmKO	cgatgaattcgagctccaccgcggtggcggccgctctagaa ctagtggatcccccgggctgcaggaccacctttgattgTTT CCCAGTCACGACGTT
CaSATDAmPmS2	CGATGAATTCGAGCTCCACCGCGGTGGCGGCCGCTCTAGAA CTAGTGGATCCCCCGGGCTGCAGGACCACCTTTGATTGtct gatatcatcgatgaattcgag
CaSATDAmPpS1	CAAGAAATCGATGGAAAGTTTCTACACTAAAGGTTATGATG AATTCCAAAGACTTACCAATGGTATTCCTTACTAgaagctt cgtacgctgcaggtc
CaSATm596	GTTTTCCCACCCTACCCAT
CaSATm1638	AATGCCGCCGAGAGTAAAG
CaSATp1315	TCTACCAGAAGTGTGAGCC
CaSATp1734	gtgaagtgtgaagggggaga
CaSATp1315	TCTACCAGAAGTGTGAGCC
CaSATp1734	gtgaagtgtgaagggggaga
CaGFPm106	ccggagacagaaaatttg
CamScarletm108	CATGACCATTCATTGAACCTTCC
CamTurq2m419	GACCTAAAATATTACCATCTTC
CaACT1m-TM	ACATTTGTGGTGAACAATGG
CaACT1p-TM	ATGTTCCCAGGTATTGCTGA
CaALS3p-TM	TTGGCAACGTGCACCTTTC
CaALS3m-TM	GTGGTCACGGCGGTAGTACTG
CaECE1p-TM	CCAGAAATTGTTGCTCGTGTTG
CaECE1m-TM	CAGGACGCCATCAAAAACG
CaEFR3m-TM	TCTTCCCATCATCTTCAACGT
CaEFR3p-TM	ACAACCCACAAAGCAGCATA

CaHGC1p-TM	AAAGCTGTGATTAAATCGGTTTTGA
CaHGC1m-TM	AATTGAGGACCTTTTGAATGGAAA
CaHWP1p-TM	CGGAATCTAGTGCTGTCGTCTCT
CaHWP1m-TM	TAGGAGCGACACTTGAGTAATTGG
CaMSS4m-TM	AATCCCTCCATCATGACCATAAA
CaMSS4p-TM	AAATCTTTTGATAAACGTGCCCTTA
CamSCARLETm-TM	TTTTTGCATAACTGGACCATCTG
CamSCARLETp-TM	GTGGTGCTGTTACTGTTACTCAAGATAC
CaPIK1αm-TM	GCTGCGATTCTCATCTGATCAGC
CaPIK1αp-TM	CCCCTTCTATTACGGAGCAACTG
CaSAC1m-TM	TTCAACACCACCTCCCAGTGT
CaSAC1p-TM	CCACAGCAACAGATGGAACTCA
CaSAP4p-TM	TCCCTTTTCCTTAACTCTCCTGAA
CaSAP4m-TM	AGCCGCTATACTTGGCCTTGT
CaSAP5p-TM	CAATTATTTGCTAACGTTTGGTCTACTAG
CaSAP5m-TM	CTTCGCCGCTTTGAAAACC
CaSAP6p-TM	TCTCCAGGGTTTGTTACCTTAGACTT
CaSAP6m-TM	TTAGATTCGGCAGTTGGATCATC
CaSTT4m-TM1	AGCCCATCAACACCATATTTTTCT
CaSTT4p-TM1	CCATTGATTTTGAAAGGGTTAAGTT
CaSTT4m-TM2	CGGTACGCAATGGTAATTTATTCAT
CaSTT4p-TM2	GATCGTGAATGGTCCGCAAT
CaTDH3p-TM	ATCCCACAAGGACTGGAGA
CaTDH3m-TM	GCAGAAGCTTTAGCAACGTG
CaYPP1m-TM	TGGTGTGGGTTGTTCTTTTTC
CaYPP1p-TM	AAATGGTCCCATCGTCCAA

References

1. Wilson RB, Davis D, Mitchell AP. 1999. Rapid hypothesis testing with *Candida albicans* through gene disruption with short homology regions. J Bacteriol 181:1868-74.
2. Vernay A, Schaub S, Guillas I, Bassilana M, Arkowitz RA. 2012. A steep phosphoinositide bis-phosphate gradient forms during fungal filamentous growth. J Cell Biol 198:711-30.
3. Ghugtyal V, Garcia-Rodas R, Seminara A, Schaub S, Bassilana M, Arkowitz RA. 2015. Phosphatidylinositol-4-phosphate-dependent membrane traffic is critical for fungal filamentous growth. Proc Natl Acad Sci U S A 112:8644-9.
4. Labbaoui H, Bogliolo S, Ghugtyal V, Solis NV, Filler SG, Arkowitz RA, Bassilana M. 2017. Role of Arf GTPases in fungal morphogenesis and virulence. PLoS Pathog 13:e1006205.
5. Bassilana M, Blyth J, Arkowitz RA. 2003. Cdc24, the GDP-GTP exchange factor for Cdc42, is required for invasive hyphal growth of *Candida albicans*. Eukaryot Cell 2:9-18.
6. Schaub Y, Dunkler A, Walther A, Wendland J. 2006. New pFA-cassettes for PCR-based gene manipulation in *Candida albicans*. J Basic Microbiol 46:416-29.
7. Silva PM, Puerner C, Seminara A, Bassilana M, Arkowitz RA. 2019. Secretory Vesicle Clustering in Fungal Filamentous Cells Does Not Require Directional Growth. Cell Rep 28:2231-2245 e5.
8. Milne SW, Cheetham J, Lloyd D, Aves S, Bates S. 2011. Cassettes for PCR-mediated gene tagging in *Candida albicans* utilizing nourseothricin resistance. Yeast 28:833-41.
